# Supplementary material for: Online images amplify gender bias
Source: Nature. 2024 Feb 14;626(8001):1049–55. doi: 10.1038/s41586-024-07068-x (PMC10901730; doi:10.1038/s41586-024-07068-x)
Supplement: Supplementary file 1 — Supplementary Information [file 41586_2024_7068_MOESM1_ESM.pdf]

nature portfolio

https://doi.org/10.1038/s41586-024-07068-x

Supplementary information

Online images amplify gender bias

In the format provided by the authors and unedited

Supplementary Appendix for:
Online Images Amplify Gender Bias

Douglas Guilbeault[1*], Solène Delecourt[1], Tasker Hull[2], Bhargav Srinivasa Desikan[3], Mark Chu[4], Ethan Nadler[5]

[1]*Haas School of Business, University of California Berkeley, Berkeley, USA*
[2]*Psiphon Inc., Toronto, Canada*
[3]*Institute For Public Policy Research, United Kingdom*
[4]*School of the Arts, Columbia University, USA*
[5]*Department of Physics, University of Southern California, USA*

Contents

Supplementary Analyses (Observational) **1**

A.1.1 Replication using Data from Wikipedia 1
A.1.2 Robustness to Analyzing Ground Truth Measures of Gender in Online Images from IMDb and Wikipedia 2
A.1.3 Robustness to Explicitly Gendered Searches 3
A.1.4 Robustness of Results to Search Location 6
A.1.5 Robustness to the Demographics of Human Coders 8
A.1.6 Evaluating the Accuracy of Human Coders 8
A.1.7 Controlling for Intercoder Agreement on Gender Classification 10
A.1.8 Robustness to Comparing Against a Word2vec Model Trained on a Recent Sample of Online News 11
A.1.9 Robustness to Alternative Word Embedding Models 12
A.1.10 Robustness to Normalization Procedure 16
A.1.11 Robustness to the Number of Categories Examined 16
A.1.12 Replication using only Categories that have the Same Gender Association across All Modalities when Comparing Images and Text 17
A.1.13 Robustness to Machine Learning Classifications 18
A.1.14 Robustness to Controlling for Linguistic Properties of Social Categories 22
A.1.15 Robustness to Alternative Constructions of the Gender Dimension 25
A.1.16 Robustness to Search Frequency in Google Images 29
A.1.17 Robustness to the Number and Ranking of Images in Google Image Search Results 31
A.1.18 Robustness to Evaluating Human Judgments of Uncropped Images 35
A.1.19 Validation of OpenCV Cropping Algorithm 37
A.1.20 Controlling for the Number of Faces in each Image, the Number of Times each Image Repeats across Searches, and whether Images depict Avatars 37
A.1.21 Robustness to the number of images associated with each search 39
A.1.22 Extended Analysis of Gender Associations in Images and Text as Compared to Census Data 39

Supplementary Analyses (Experimental) **41**

A.2.1 Robustness to Participants Searching in Google Instead of Google News 41
A.2.2 Robustness of Experimental Results to Controlling for Time and the Number of Sources 42
A.2.3 Robustness to Controlling for Participants' Gender 45
A.2.4 Using our Observational Measures of Gender Bias to Predict Gender Bias in Participants' Uploaded Descriptions and Self-report Beliefs 47
A.2.5 Robustness to Controlling for Experimental Condition (Fig. 3CD) 49
A.2.6 Comparing the Strength of Gender Priming between Text and Images 49
A.2.7 Robustness to Statistical Controls (Fig. 3E) 51
A.2.8 Experimental Effects of Images on Implicit Bias Over Time 52
A.2.9 Examining the Sources of Online Images 54

Supplementary References **55**

Supplementary Analyses (Observational)

A.1.1 Replication using Data from Wikipedia

Here, we illustrate that our main analyses replicate using data from Wikipedia. To measure gender associations in the texts of Wikipedia articles, we apply the same gender dimension method described in the main text to a pre-trained word embedding model of Wikipedia available in Python's gensim package, which was built using the GloVe method [1] to analyze a 2014 corpus of 5.6 billion words from Wikipedia. A strength of using gensim's pre-trained embeddings of Wikipedia is that gensim provides pre-trained versions of the same model while changing the dimensionality, including a 50-D, 100-D, 200-D and 300-D version. This allows us not only to test whether our results hold over Wikipedia, but also to test whether these results are robust to varying the dimensionality of the word embedding model. This is an important robustness test for ruling out the concern that our findings regarding the differences between images and text are driven by the significantly greater dimensionality of textual measures, which could allow this approach to detect finer associations; instead, we find that reducing the dimensionality of our textual model has no impact on our results.

To measure gender associations in images over Wikipedia, we use each social category from Wordnet (n = 3,495) to search for Wikipedia articles focusing on these social categories. For example, we retrieve the Wikipedia article with the title 'Physician' for the social category *physician*: https://en.wikipedia.org/wiki/Physician. After identifying those social categories with corresponding Wikipedia articles, and after identifying which among these categories were also contained within our word embedding model of Wikipedia, we were left with a dataset of 1,244 social categories, including both occupations (e.g. *physician*) and social roles (e.g. *passenger*). We then applied the same auditing framework to represent a category's gender associations in images according to Wikipedia (see "Main Data Collection Procedure"). For each category, we extracted the publicly available images that Wikipedia provides on the Wikipedia article corresponding to this category. All images from Wikipedia were extracted using WIT ("Wikipedia Image Text Dataset), one of the largest multimodal datasets on record (to date) [2]. Gender associations in both texts and images over Wikipedia were represented along a -1 (female) to 1 (male) axis, where -1 indicates 100% female associations and 1 indicates 100% male associations (see "Constructing a Gender Dimension in Word Embedding Space"). On average, we identified 7 images per social category (SD = 7 images) over Wikipedia, and on average these 7 images yielded 12 faces per category (SD = 17). For robustness, we only examine categories that are associated with at least ten faces in the Wikipedia data, leaving 495 categories for analysis. All of the following results equally hold if we include all 1,244 categories in our analysis (we also report these results below).

Extended Data Fig. 1 shows the results of applying our auditing framework to image and text data from Wikipedia. Panel A of Extended Data Fig. 1 demonstrates that the absolute strength of gender associations is significantly higher in images (μ = 0.33), as compared to word embedding models of Wikipedia, regardless of their dimensionality (p < 0.0001, Wilcoxon signed-rank test, two-tailed; all word embedding models exhibit an average strength of gender association below 0.1). Altering the dimensionality of the word embedding models has no qualitative effect on the distribution of gender associations.

Further consistent with our analysis of Google, we find that images over Wikipedia are significantly skewed toward male representation. 80% of categories are male-skewed according to images over Wikipedia ($p < 0.0001$, proportion test, $n = 495$, two-tailed), whereas word embedding models of Wikipedia with different dimensionality show, respectively, 57% (50D), 59% (100D), 57.6% (200D), and 54% (300D) of categories as male-skewed, all of which are significantly lower than Wikipedia images at the $p < 0.0001$ level (proportion test, two-tailed). Including all 1,244 categories in our analysis continues to show a strong bias toward male representation in Wikipedia images (with 68% of faces being male, $p < 0.00001$). This bias is substantially higher than all Wikipedia word embedding models of all 1,244 categories at the $p < 0.0001$ (50D = 52%, 100D = 53%, 200D = 53%, 300D = 50%). The average strength of gender association in the images linked to all 1,244 categories ($\mu = 0.28$) is also significantly higher than all Wikipedia embedding models of these same categories at the $p < 0.0001$ level (all word embedding models exhibit an average strength of gender association below 0.06).

These results are striking in light of the popular belief that Wikipedia is a neutral source of information [3]. Wikipedia content can appear to be neutral in its gender associations if one focuses only on text, whereas examining Wikipedia images from the same articles can reveal a different reality, with evidence of a strong bias toward male representation and a stronger bias toward more salient gender associations in general.

A.1.2 Robustness to Analyzing Ground Truth Measures of Gender in Online Images from IMDb and Wikipedia

In this section, we validate our theory using the IMDb-Wiki dataset [4], which contains over half a million online images of celebrity faces for which the ground truth gender of the face is known via the celebrity's public biographical page on either IMDb or Wikipedia. This dataset contains 460,723 faces of celebrities from IMDb and 62,328 faces of celebrities from Wikipedia (covering 72,214 celebrities in total); each of these faces is associated with the self-identified gender of the celebrity in the image. As described by Rothe et al. (2018) [4], this dataset was compiled by, first, identifying the top 100,000 celebrities according to IMDb (this dataset includes actors, as well as producers, directors, public figures, athletes, and more, such that "celebrity" is the most appropriate category of reference for this data, as the authors describe). Then, Rothe et al. (2018) [4] automatically crawled from IMDb the name, age, and gender of the celebrities from this list, as well as all images associated with each celebrity over the IMDb website. The authors additionally crawled all profile images from pages of these celebrities from Wikipedia. They removed the images without a timestamp (the date when the photo was taken). In total, they obtained 460,723 face images from 20,284 celebrities from IMDb and 62,328 from Wikipedia, thus 523,051 in total. As an additional pre-processing, all faces without an associated gender identification are removed. This resulted in 452,261 face images from 19,091 celebrities from IMDb and 59,685 face images from 58,904 celebrities from Wikipedia, and thus 511,946 faces in total.

The IMDb-Wiki dataset allows us to test our theory using true gender assignments independent of our human coders' judgments. For this analysis, we used two different word

embedding models of the Wikipedia corpus: the 300-dimensional 2014 GloVe model [1] and the 300-dimensional 2017 FastText model from Facebook [5]. In each model, we determine the gender association of the word "celebrity" using our standard method of constructing a gender dimension in word embedding space. Moreover, to identify the gender representation with the social category of 'celebrity' in the census, we used the aggregated gender representation in the census' CPS category of occupations relating to "arts, design, entertainment, sports, and media occupations," which includes the occupations "actors", "directors", "musicians", and "entertainers/performers."

Extended Fig. 2 below presents the results of comparing the gender associations with the social category "celebrity" across these five datasets: the IMDb-Wikipedia Face Image dataset (for which the true gender of each face is known), the census (for which the true empirical distribution of gender is known), the gender associations in the 2014 GloVe word embedding model of Wikipedia text, and the gender associations in the 2017 FastText word embedding model of Wikipedia text.

As panel A of Extended Data Fig. 2 demonstrates, online texts encode more female associations for the category "celebrity" as compared to the census. Specifically, the gender association of the category "celebrity" is -0.05 according to the FastText model of Wikipedia texts and -0.08 according to the GloVe model of Wikipedia texts. Meanwhile, the census indicates that 49% of people in occupations relating to celebrities are women, resulting in a gender association score of 0.02 that fails to be significantly skewed toward a particular gender (p = 0.54). By contrast, online images from Wikipedia encode substantially greater male representation compared to both the census and Wikipedia texts. The gender association of the category "celebrity" is 0.57 according to Wikipedia images. The same data is shown for IMDb images of celebrities, which are also significantly more biased toward male representation (0.16 along the gender scale) compared to these textual measures and the census. Panel B of Extended Data Fig. 2 indicates that this skew toward male representation in online images is highly significant; over IMDb, 58% of celebrity images are of men (p < 0.001, proportion test, n = 482,261), and over Wikipedia, 79% of celebrity images are of men (p < 0.001, proportion test, n = 59,681). These results strongly support our theory using online images that are associated with verified ground truth gender classifications, indicating that our findings hold independently of the subjective gender judgments of human coders.

A.1.3 Robustness to Explicitly Gendered Searches

As an additional analytic strategy for identifying male bias in Google Images, we evaluate whether Google is more effective at retrieving male as opposed to female faces for each category when it is explicitly asked to do so. For this test, we collect and analyze the top 100 images from Google when searching with each category on its own (e.g. *doctor*), and also when searching with each gender labeled explicitly (e.g. *female doctor* and *male doctor*). We only used categories that were identified as explicitly non-gendered (e.g., excluding categories such as *girl*), leaving us 3,084 categories, which yielded 491,169 images. See Fig. S1 for an overview of this methodology. The images in this analysis were coded by the same team of 6,392 coders reported in our main study, using the same methodology whereby each image was coded by three unique coders. Identical to our main analyses, each search was

implemented from a fresh Google account with no prior history to avoid the uncontrolled effects of Google's recommendation algorithm which customizes search results based on users' browsing history (searches were run by 10 distinct data servers in parallel from New York City).

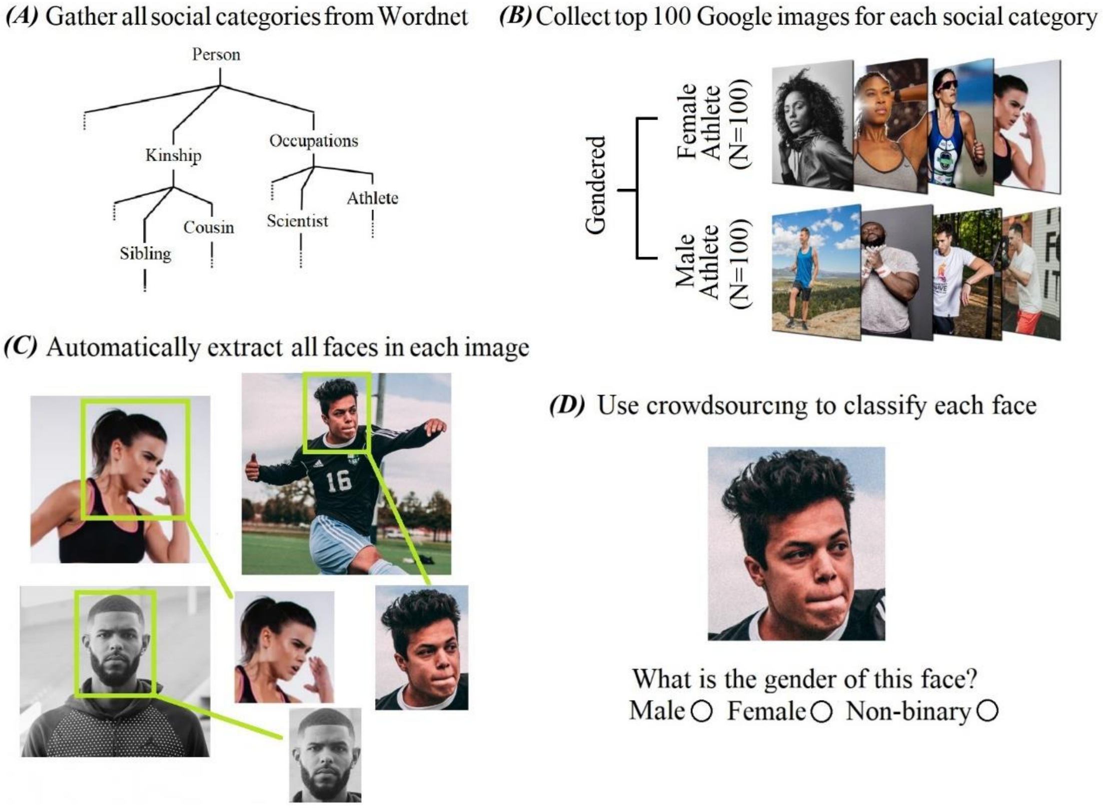

Fig. S1: A schematic diagram of the data collection methodology used for explicitly gendered searches. The method described here is the same as the method applied for the results described in the main text.

Using this data, we examine whether the Google search engine is more successful at retrieving male rather than female examples of the same social category when explicitly asked to do so. Specifically, for each category, we calculate the probability that a male-specific search returns a male face, and from this we subtract the probability that a female-specific search with the same category returns a female face. Positive values indicate that male-specific searches were more effective at returning male faces than female-specific searches were at returning female faces, for the same category.

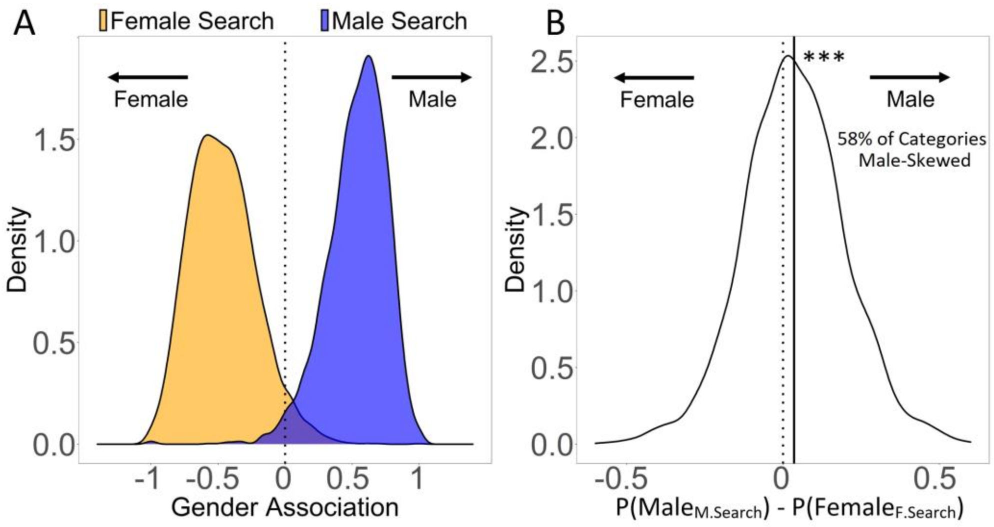

Fig. S2: (A) The distribution of gender associations for 3,096 categories in Google Images, shown separately for female- and male-specific searches of the same category (e.g., searching "male doctor" and "female doctor" separately; fig. S1). (B) The bias score for each category is determined by subtracting the probability that a female-specific search returns female faces from the probability that a male-specific search returns male faces, for the same category (positive values indicate a bias toward male representation). ***, $p < 2.2 \times 10^{-16}$ (t-test, two-tailed).

Panel A of Fig. S2 confirms, as expected, that female-specific searches yielded significantly more female search results, while male-specific searches for the same categories yielded significantly more male search results. However, panel B of Fig. S2 shows that the ability for Google to successfully provide male faces in response to male-specific searches is significantly better than its ability to provide female faces in response to female-specific searches ($p < 0.0001$, Wilcoxon signed rank test, two-tailed). Indeed, for 58% of categories, Google was more successful at retrieving male than female faces for gender-specific searches, exhibiting significant male bias ($p < 0.001$, proportion test, two-tailed). Thus, even when requesting equally female and male search results, we continue to observe a significant bias toward the over-representation of men in Google Image search results.

Consistent with these findings, we also show that when Google provides the wrong gender for a gendered query – e.g., by showing a female when searching for a "male doctor" or showing a male when searching for a "female doctor" – such errors are significantly more likely to favor male representation. Men are significantly more likely to mistakenly appear in female-specific searches (27%) than women are to appear in male-specific searches (23%), ($p < 0.001$, $n = 2{,}960$ categories, t-test, two-tailed). This error rate significantly favors male representation for 58% of categories ($p < 0.001$, proportion test, two-sample).

A.1.4 Robustness of Results to Search Location

Google is known to tailor search results to IP location [6]. Here, we show that our results hold when collecting Google images from 5 additional IPs from around the world. We tested the generality of our Google results by analyzing the top 100 images associated with 300 categories when searching from six distinct locations: Singapore (the Republic of Singapore), Frankfurt (Germany), Bangalore (India), Toronto (Canada), and Amsterdam (the Netherlands). These IP locations were selected based on those available through DigitalOcean, a public VPN provider. All searches were run during the same time window. The 300 categories comprised 256 occupations that could be mapped between our WordNet dataset and the US census data, along with a random selection of additional categories (n = 44). The gender of images in this replication were classified using the same procedure in our main study, whereby the final gender classification associated with each face was determined by the modal (majority) gender classification across three unique coders. We hired 1,223 annotators to categorize these images. Again, all coders were based in the US and were fluent English speakers.

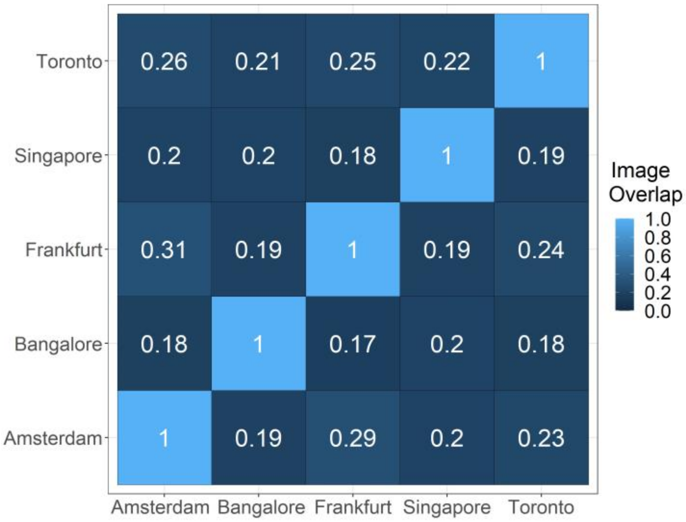

Fig. S3: Comparing the Google images across search locations (IPs) from distinct countries, namely: (i) New York (USA), (ii) Singapore (the Republic of Singapore), (iii) Frankfurt (Germany), (iv) Bangalore (India), (v) Toronto (Canada), and (vi) Amsterdam (the Netherlands). This figure shows the average overlap in the images that appeared for the same search queries across all search locations. Overlap is measured via the Jaccard Index, defined as the proportion of common elements between two sets compared to the total number of unique elements in both sets.

First, we examine the extent to which the image search results across IPs were similar for the same searches. We conducted this analysis by using image metadata and pixel level comparisons to identify whether an image repeated across searches. Consistent with

location-specific search results, Fig. S3 estimates that on average, only 21% of the image search results were the same across locations. Any consistency in the patterns of gender bias across these sources cannot, therefore, be attributed to stability in Google's search results across IPs. Instead, these results indicate that changing the geolocation of one's IP produces qualitatively distinct image search results in Google.

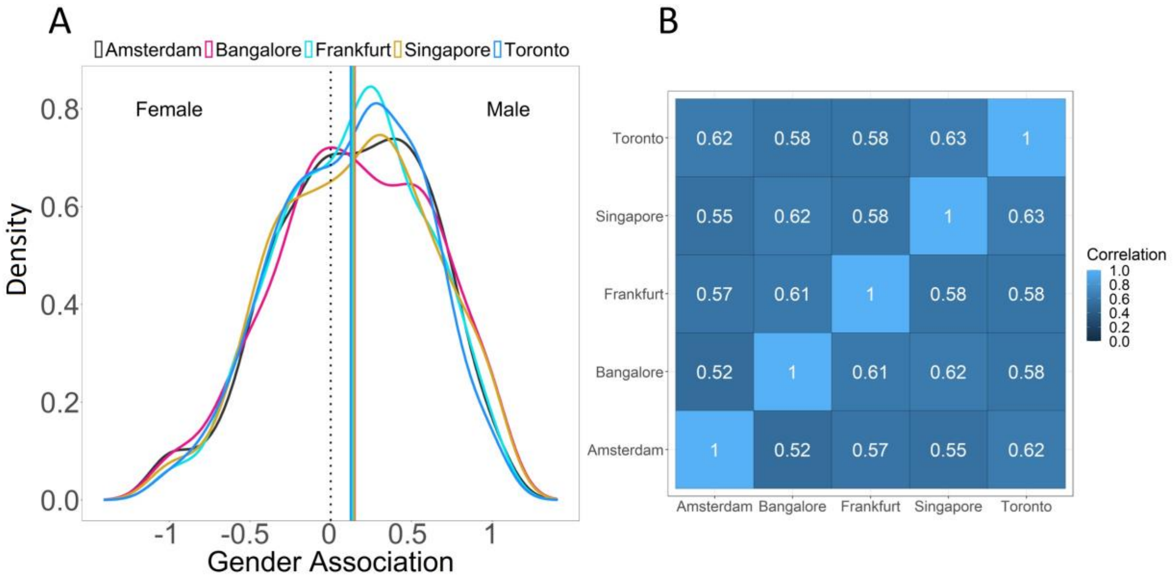

Fig. S4: (A) The distribution of gender associations for 300 social categories in Google Images collected via online searches from five distinct locations (IPs) from distinct countries, namely: (i) Singapore (the Republic of Singapore), (ii) Frankfurt (Germany), (iii) Bangalore (India), (iv) Toronto (Canada), and (v) Amsterdam (the Netherlands). The image-based measure captures the frequency of male and female faces associated with each category in Google Image search results (-1 means 100% female; 1 means 100% male). Vertical lines display the average gender associations across categories for each IP; the dotted vertical line indicates perfect gender balance (0 along the gender dimension). (B) The pairwise correlations in the gender associations of categories as they appear in Google images collected via online searches from these distinct IPs.

Despite the differences in images returned by Google searches from different IPs, we nevertheless find strikingly similar patterns in the gender associations of categories in these distinct image sets. Fig. S4 shows that, for all IPs, the categories examined skew significantly toward male representation in Google images ($p < 0.001$ for all IPs, Wilcoxon signed-rank test, two-tailed). Moreover, the gender bias displayed in the Google Image search results across these IPs is significantly stronger than the measure of gender bias according to word embedding models of Google News, as reported in the main text ($p < 0.001$ for all IPs, Wilcoxon signed-rank test, two-tailed). Panel B of Fig. S4 displays the pairwise correlation in

the gender associations of categories as they appear in Google images from these distinct IPs. The gender association by categories is highly and significantly correlated across IPs ($p < 0.001$ for all pairwise comparisons measured using either Pearson or Spearman correlation).

A.1.5 Robustness to the Demographics of Human Coders

One potential concern is whether the demographic composition of our annotator panel may lead to biases in how they classify the faces in our dataset. Here, we report the results of a logistic regression that predicts the gender of the face retrieved by Google Image search, while controlling for the demographic features of the Mturk workers who coded the faces (in addition to including random effects for each Mturk worker).

Table S1 presents the results of a logistic regression predicting the identified gender of a face (male or female), with random effects for the social category, as well as fixed effects to control for the demographics of the human coders themselves, including their age, race, gender, education level, yearly income, and political party. Table S1 indicates that, even when controlling for image, search term, human coders, as well as the demographics of human coders, the main result still holds strongly that male faces are 1.53 times likely to be returned when searching in Google than female faces ($p < 0.001$). Importantly, the fixed effects associated with the demographic traits of Mturk workers were unrelated to the expected gender classification of faces, contributing to a marginal R^2 of only .019, while the model as a whole achieves a conditional R^2 of 0.186. This robustness analysis indicates that our results are unlikely to be driven by demographic-related biases in the gender classifications provided by our annotator pool.

A.1.6 Evaluating the Accuracy of Human Coders

To further evaluate the accuracy of the gender judgments of our human coders, we developed a separate validation task where a subset of coders classified faces for which the true age and gender of each face is known. For this purpose, we used the IMDb-Wiki image dataset [4], which maps the birth-date and gender of celebrities (according to their IMDb and Wikipedia profiles) to time-stamped photos depicting these celebrities in order to infer the age of the celebrity as captured at the time of each photo. A strength of this dataset is that it contains over 500k faces, with substantial representation across all age groups. Other canonical image datasets are less helpful for this test, since they lack coverage across age groups. For example, the Chicago Face dataset [7], a commonly used repository of images, only contains faces between 18 and 40 years old. Another popular dataset for evaluating human gender and age judgments does not contain any infants, children, adolescents, or elderly people [8].

	Gender of Face [Male]			
Predictors	*Odds Ratios*	*std. Error*	*CI*	*p*
(Intercept)	1.53	0.12	1.31 – 1.80	**<0.001**
coder age [18-24]	0.84	0.06	0.73 – 0.98	**0.023**
coder age [25-34]	0.92	0.07	0.80 – 1.07	0.268
coder age [35-54]	0.89	0.07	0.77 – 1.04	0.136
coder age [55-74]	0.83	0.07	0.70 – 0.97	**0.021**
coder income [Less than 10k]	0.95	0.01	0.93 – 0.98	**0.002**
coder income [10k-50k]	1.02	0.01	0.99 – 1.05	0.124
coder income [50k-75k]	0.97	0.01	0.94 – 0.99	**0.013**
coder income [75k-100k]	1.02	0.02	0.99 – 1.05	0.134
coder income [150k-250k]	0.85	0.02	0.81 – 0.89	**<0.001**
coder income [Over 250k]	1.07	0.05	0.98 – 1.17	0.153
coder income [Rather not say]	0.30	0.03	0.25 – 0.36	**<0.001**
coder education [Doctorate (PHD)]	0.90	0.04	0.83 – 0.99	**0.030**
coder education [High School]	0.59	0.01	0.56 – 0.62	**<0.001**
coder education [Master's]	0.92	0.02	0.88 – 0.97	**0.001**
coder education [Rather not say]	0.84	0.22	0.51 – 1.39	0.494
coder education [Tech./Comm. College]	0.79	0.02	0.76 – 0.83	**<0.001**
coder education [Undergraduate]	0.93	0.02	0.89 – 0.97	**0.001**
coder political ideo. [Conservative,Other]	2.79	0.54	1.91 – 4.07	**<0.001**
coder political ideo. [Independent]	1.03	0.01	1.01 – 1.05	**0.009**
coder political ideo. [Liberal]	1.03	0.01	1.01 – 1.05	**0.002**
coder political ideo. [Liberal,Conservative]	0.60	0.51	0.11 – 3.21	0.551
coder political ideo. [Other]	0.98	0.03	0.92 – 1.03	0.408
coder political ideo. [Rather not say]	1.02	0.03	0.97 – 1.07	0.408
coder gender [Male]	1.12	0.01	1.10 – 1.14	**<0.001**
coder gender [Non-binary]	0.68	0.01	0.65 – 0.70	**<0.001**
coder gender [Rather not say]	1.09	0.05	1.00 – 1.19	0.058
coder race [Asian]	1.17	0.02	1.14 – 1.21	**<0.001**
coder race [Caucasian]	1.21	0.02	1.18 – 1.25	**<0.001**
coder race [Hispanic]	1.00	0.02	0.96 – 1.03	0.886
coder race [Native American]	1.15	0.02	1.12 – 1.19	**<0.001**
coder race [Native Hawaiian]	1.19	0.06	1.08 – 1.31	**<0.001**
coder race [Other]	1.01	0.07	0.88 – 1.16	0.907
coder race [Rather not say]	1.09	0.05	0.99 – 1.20	0.087
coder race [Two or more]	1.15	0.07	1.02 – 1.29	**0.019**
coder race [Two or more races]	1.70	0.14	1.44 – 2.00	**<0.001**
**Random Effects**				
σ^2	3.29			
τ_{00} Social.Category	0.68			
ICC	0.17			
N Social.Category	3457			
Observations	352,937			
Marginal R^2 / Conditional R^2	0.019 / 0.186			

Table S1: Logistic Regression (Binomial, Mixed Model) predicting the identified gender of a face (male or female), with random effects for the social category (Social.Category), as well as fixed effects to control for the demographics of the human coders themselves, including their age, race, gender, education level, yearly income, and political party. Random effects by social category were employed in this setting since, by design, the relationship between coders' demographics and the categories of images they encountered was random.

For this validation task, we selected 5 male and 5 female faces from the IMDb-Wiki dataset across seven age bins, totaling 70 unique images. The age bins were: 0-11, 12-17, 1824, 25-34, 35-54, 55-74, and +75. We then recruited a random sample of 215 human coders from our original coder sample to classify the gender of each of these faces. Each coder classified 35 images randomly sampled from the entire set of 70. The order of images in this classification task was randomized. To control for familiarity biases, we asked each coder to indicate whether they were familiar with the face being classified. Only 5% of responses indicated that a participant was familiar with a particular face. There was no significant difference in the extent to which participants indicated familiarity as a function of the gender of the face (p = 0.11, Wilcoxon rank sum test), nor as a function of the age group of the face (p = 0.88, JT = 1036, Jonckheere-Terpstra test). Our main results control for participants' familiarity judgments, and all of our main results hold if we exclude participant judgments that indicated familiarity with the face being classified. On average, across all age groups, coders were able to accurately identify the self-identified gender of the person depicted 97% of the time, with no significant differences in this accuracy rate across age groups. These results lend further confidence to the gender judgments provided by our human annotators.

A.1.7 Controlling for Intercoder Agreement on Gender Classification

Another important indicator of data quality in crowdsourced human judgments is intercoder agreement. We follow prior work by hiring three unique human coders to classify each face [9,10]. Across all images in our Google dataset, coders reached 91% agreement in their gender classifications on average, which is considered high based on standard measures of intercoder reliability [11]. The rate of intercoder agreement did not significantly vary as a function of the modal gender identified for a given face (t-test, $p < 0.52$, two-tailed).

For robustness, we also calculate the chance-corrected inter-coder reliability of raters in our sample using GWET's AC [12]. We calculated GWET's AC using the irrCAC package in R. Our coders achieved a coefficient of 0.48. This GWET coefficient falls within the 'good' range of reliability according to standard interpretations of this measure, especially considering that our sample was limited to only three coders per image (see [12]). Combined with our percent-agreement results, these analyses suggest that our coders provided reliable statistical judgments of the gender of faces in our sample.

	Coefficients	Odds Ratio
Intercoder Agreement	-0.01	0.99
	(0.02)	
Constant	0.10***	1.12***
	(0.02)	
N	703,166	
R^2	0.00	

Standard errors in parentheses (clustered by social category)
* $p < 0.05$, ** $p < 0.01$, *** $p < 0.001$

Table S2: Logistic Regression (binomial) predicting the modal gender identification of a face (male or female), while controlling for the rate of intercoder agreement in this

model classification among annotators. Standard errors are clustered by social category. Model includes all Google images in our dataset, including the Google images gathered by searching for gender-specific search results (e.g., by searching for "male doctor" and "female doctor" separately).

For additional robustness, we use a logistic regression to predict the likelihood of observing a male face in our entire image dataset, controlling for the rate of intercoder agreement associated with each face. Table S2 displays the results of this model. We find that the rate of intercoder agreement on gender classification fails to significantly predict the likelihood of observing a male image (OR = 0.99 p = 0.71); meanwhile, we find that men continue to be 1.12 times more likely to appear in our Google image dataset, controlling for the rate of intercoder agreement associated with each image ($p < 0.0001$).

We further show that intercoder agreement similarly holds when controlling for the gender of the coders in our sample. Here, we examine the relationship between coder gender and the gender classification provided by coders for all images collected from Google, including those associated with gender-specific searches (since this maximizes the statistical power of this analysis). Female coders classified 49.9% of images as female, marking no significant bias toward same gender classification (p = 0.94, proportion test, two-tailed). Male coders classified 52.9% of images as male, marking a weak bias toward same gender classification ($p < 2.2 \times 10^{-16}$, proportion test, two-tailed).

52.2% of images as female, and male coders classified 48.1% of images as female. While this distribution suggests that coders are slightly biased toward classifying faces in accord with their own gender, this effect is weak; both male and female coders classified faces as male or female at a roughly 50/50 rate. Importantly, we show that our main results are robust to controlling for the demographics of annotators (see "Robustness to the Demographics of Human Coders").

A.1.8 Robustness to Comparing Against a Word2vec Model Trained on a Recent Sample of Online News

One potential concern for our main analyses is that the data on which the canonical word2vec model is trained on is from 2013 [13], while our Google Image data was collected between 2020 and 2021. This raises the concern that perhaps the differences we observe between these text-based and image-based measures are driven by the discrepancy in *when* the data was collected, not in the modality of the data (image vs. text). To address this concern, we trained our own word2vec model on a more recent sample of online news data published between 2021 and 2023. We compiled a dataset of 2,717,000 randomly sampled news articles published in English across various topics between January 2021 and August 2023. These articles were sourced from the following prominent online news sources: 1,000,000 articles from the BBC; 500,000 articles from the Huffington Post; 480,000 articles from CNBC; 400,000 articles from Bloomberg; 160,000 articles from Time Magazine; 150,000

articles from Techcrunch; and 27,000 articles from CNN. These datasets were purchased from the online web-scraping service, Crawl Feeds (https://crawlfeeds.com/). We trained our own word2vec model on this dataset of online news using the exact specifications of the original word2vec model trained on the Google News dataset. Specifically, our word2vec model used 300 dimensions with skip-gram training based on a window size of 10. We then apply the same method we applied to the Google News word2vec model for identifying the gender associations of all social categories in Wordnet. The aim of comparing against this retrained word2vec model is to show that our main results are not an artifact of the time difference between the textual data collection for training the original Google News word2vec model (2013) and the time-frame during which we collected our Google Image data (2021).

First, we show that the gender associations of the social categories in Wordnet are remarkably consistent across the 2013 Google News word2vec model and our own word2vec model, which was retrained on a sample of online news from 2021 to 2023. Panel A of Extended Data Fig. 4 shows that the Pearson correlation in gender association across these models, paired at the category level, is 0.79 and highly statistically significant ($p < 0.00001$). Accordingly, the gender associations in the 2013 Google News word2vec model and our 2023 retrained word2vec model are both highly correlated with the gender associations captured in our main Google Image sample, both yielding a Pearson correlation ($p < 0.0001$). Panel B of Extended Data Fig. 4 displays the overall strength of the gender associations in both word2vec models, as compared to our main Google Image sample. There is no significant difference in the strength of gender associations between the 2013 word2vec model (μ = 0.22) and our 2023 retrained word2vec model (μ = 0.22) (p = 0.14, t-test, two-tailed). However, the strength of gender associations in our Google Image data (μ = 0.39) is significantly higher than that of both word2vec models ($p < 0.00001$, t-test). Together, these analyses provide strong evidence that our main results are not driven by the temporal difference in data collection between the 2013 Google News word2vec model and our 2021 dataset of Google images. Moreover, these analyses suggest that gender associations for social categories have remained relatively stable in online news over the last decade, a trend consistent with recent work examining gender bias in Google books [14,15]. In the next supplementary section, we contextualize these results within a broader permutation analysis, which demonstrates the robustness of our findings across alternative word embedding models employing different data sources and algorithmic specifications.

A.1.9 Robustness to Alternative Word Embedding Models

Here, we show that our main results equally hold when comparing our Google Image data against a wide range of state-of-the-art word embedding models. These models vary along a rich array of methodological parameters, including window size, data sources, the date of the data collection (ranging from 2013 to 2023), along with the algorithms they use to generate word vectors based on the distribution of words and characters. Table S3 describes each embedding model we compare against. All models are trained on online textual corpora that contain many billions of word tokens.

Model	Paper	Year	Window Size	Dimensions	Datasets	Size
word2vec-google news	Mikolov et al. (2013)	2013	10	300	-Google News (2013)	-100 billion word tokens.
word2vec-online news	Guilbeault et al. (2023)	2023	10	300	-1,000,000 articles from BBC<br>-500,000 from Huffington Post<br>-480,000 from CNBC<br>-400,000 from Bloomberg;<br>-160,000 from Time<br>-150,000 from Techcrunch<br>-27,000 from CNN.	-2 billion word tokens.
glove-gigaword	Pennington et al. (2014)	2014	10	50, 100, 200, 300*	-Wikipedia (2014)<br>-Gigaword News Archive (2003)	-5.6 billion word tokens.
glove-twitter	Pennington et al. (2014)	2014	10	50, 100, 200, 300*	-Twitter (2014)	-27 billion word tokens.
fasttext-wiki-news-subwords	Mikolov et al. (2018)<br>*Facebook	2017	5	300	-Wikipedia (2017)<br>-UMBC<br>-Statmt.org	-16 billion word tokens.
Conceptnet 5.5	Speer et al. (2017)	2017	Ensemble of wordnet, glove, and conceptnet	Ensemble	-ConceptNet Graph,<br>-Google News (2013, word2vec)<br>-Wikipedia (2014, glove)<br>-OpenSubtitles 2016	-Full corpora for 10 languages.
CharacterBERT	Boukkouri et al. (2020)<br>*Google	2020	NA	768	-Wikipedia (2020)<br>-Brown Corpus<br>-OpenWebText	-2.14 billion word tokens from Wikipedia and<br>-1.2 billion tokens from crawled websites.
GPT3	Brown et al. (2020)<br>*OpenAI	2020	NA	Estimated at 12,288	-Common Crawl<br>-WebText2<br>-Books1<br>-Books2<br>-Wikipedia	-Common Crawl: 410 billion tokens<br>-WebText2: 19 billion tokens<br>-Books1: 12 billion tokens<br>-Books2: 55 billion tokens<br>-Wikipedia: 3 billion tokens

Table S3: Summary of the word embeddings models examined in this study.

Table S3 reveals the range of model designs leveraged in our analysis. For example, we examine word2vec [13] and GloVe models [1] which use the same window size but which vary in the online sources of text they examine (including Google News, Wikipedia, and Twitter). Given that the word2vec model we examine in our main results was trained on data from 2013, we also replicate our analyses when comparing against a word2vec model trained to the identical specifications on a dataset of online news published between 2021 and 2023 (see fig. S7). We also evaluate our results using Facebook's FastText embeddings [5], which vary from prior models by using a window size of 5, while also relying on a more recent sample of Wikipedia from 2017. We further examine our results using embeddings from ConceptNet [16], which is an ensemble approach to building word embeddings that combines word2vec and Glove, along with various sources of human crowdsourcing, manual lexical ontology construction across 300 unique languages, and a large 2016 sample of online news. We also evaluate our theory using a fundamentally different kind of model – CharacterBERT [17] – which generates word embeddings by examining co-occurrence patterns at the level of characters as well as words (n-grams); this model tracks full character sequences at the level of both words and sentences in a contextually-dynamic manner, such that window size varies at the word and sentence level and is therefore not a fixed contextual window like the other models. What is more, CharacterBERT is trained on a more recent 2020 Wikipedia dataset, as well as a comprehensive 2020 sample of scraped texts from public

websites all over the open web. Lastly, we compare our results against a recent, super-large language model, GPT-3 [18], which uses a novel approach ("Generative Pre-trained Embeddings") and which possesses substantially more dimensions compared to other models, as well as a flexible and large window size; this method relies on textual data from a combination of Wikipedia, online books, and a representative random sample of websites known as the common crawl.

For all word embedding models, the gender associations of each category were calculated using exactly the same methodology we applied to the Google News corpus in the main text [19]: (1) a one-dimensional gender vector is constructed consisting of two poles, the 'male' and 'female' pole; the male pole consists of words in vector space relating the men, such as *male, man, he, and his*, and the female pole consists of the mirror feminine terms, such as *female, woman, her, and hers*; (2) then, to identify the gender association of each category of interest, one calculates the average pairwise cosine distance between this category and each gender pole, with the effect of locating this category along this gender dimension, ranging from -1 (female) to 1 (male). Where a social category falls along this gender dimension captures the frequency with which this category co-occurs with words relating to either women or men. To maximize the correspondence between our text-based and image-based measures, we apply min-max normalization separately to each text-based model of gender associations (i.e., in Wikipedia, Twitter, and Google News), so that -1 and 1 represent the most female and male categories respectively according to each measure.

In what follows, we show that our core results equally hold across all of these diverse models. In this way, we show that our results are robust to varying the core aspects of word embedding models, including window size, data source, time of data collection, and algorithmic method for generating embeddings.

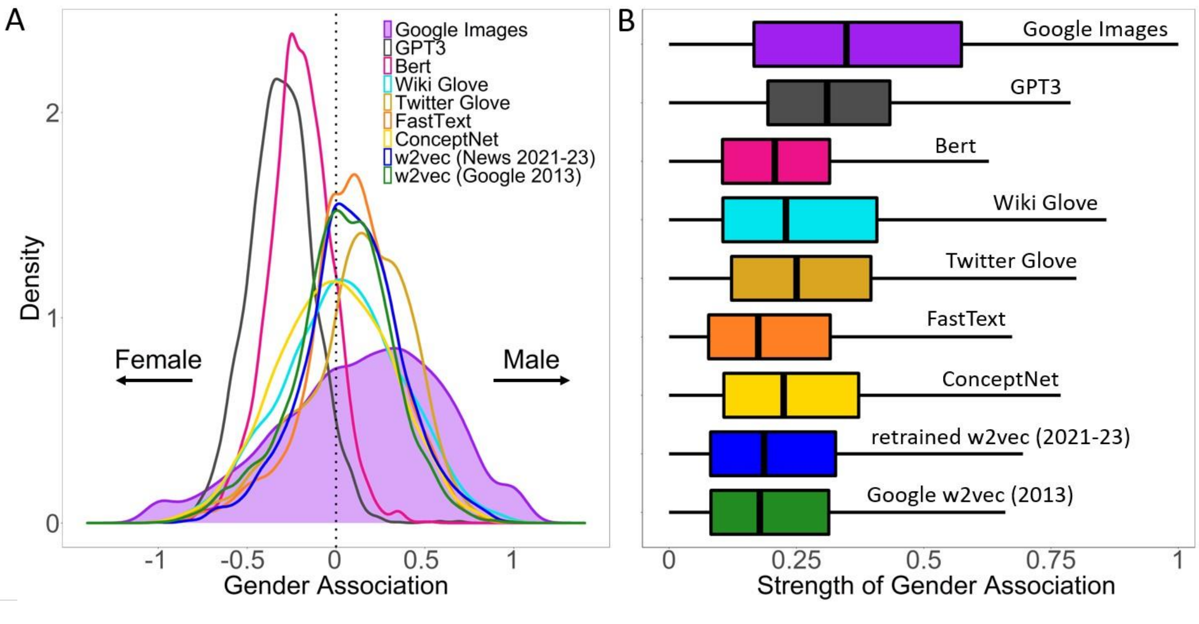

Fig. S5: (A) The distribution of gender associations for all matched categories (n = 3,426), according to all models. (B) The overall strength of gender association for all

matched categories (n = 3,426), according to all models. See Table S3 for a full description of each model.

Fig. S5*A* shows that the gender association in Google images are significantly more male than all language models examined (all comparisons between our image data and each word embedding model are significant at the $p < 0.0001$ level; Wilcoxon signed-rank test, two-tailed). In fact, both GPT-3 and BERT exhibit significant biases toward female associations, in contrast to male over-representation in online images. Fig. S5*B* further shows that the absolute strength of the gender associations in Google images is also significantly stronger than all language models (all comparisons between our image data and each word embedding model are significant at the $p < 0.0001$ level; Wilcoxon signed-rank test, two-tailed). Both of these findings are much stronger if we compare our image data against the non-normalized distribution of gender associations in each word embedding model. This is especially true when comparing against the non-normalized embeddings from GPT-3 and BERT, which have the highest dimensionality and exhibit weak differentiation of categories along the gender dimension in their raw gender associations. The weak gender differentiation of categories in GPT-3 and BERT is likely related to the fact that our analyses examine the static base embeddings of these models [20–22]. Since we do not examine the contextualized embeddings of BERT and GPT-3, their skew toward female representation according to their base embeddings may not hold once these static embeddings are contextualized [20–22].

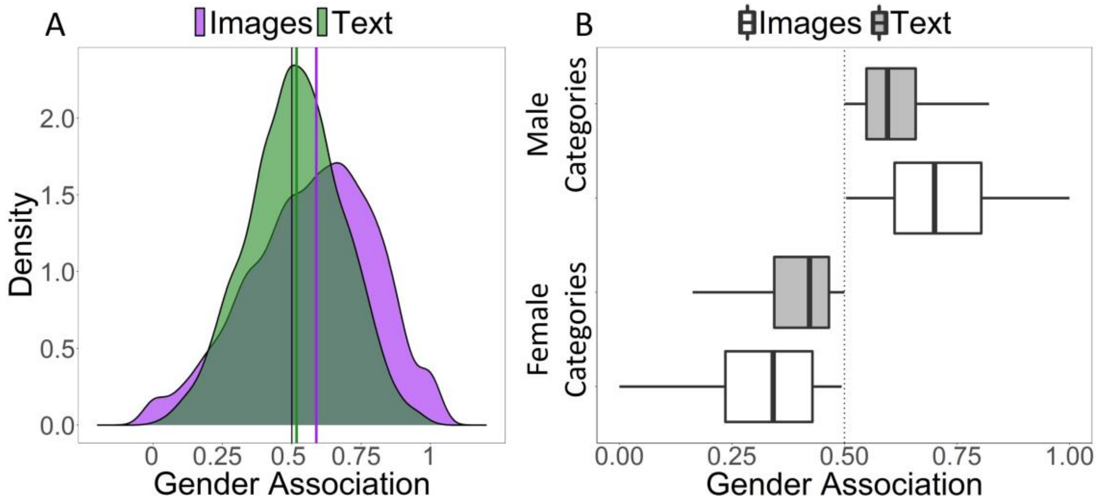

Fig. S6: A replication of Fig. 2 from the main text, while using an alternative technique for normalizing gender associations in text. (A) The distribution of gender associations for 2,986 social categories in Google Images and texts from Google News. The image-based measure captures the frequency of male and female faces associated with each category in Google Image search results (-1 means 100% female; 1 means 100% male); the text-based measure captures the frequency at which each category is associated with male or female terms in the Google News corpus of over 100 billion

words (-1 means 100% female; 1 means 100% male associations). Solid green (purple) line indicates the average gender association according to text (images). (B) The strength of gender association according to these texts and images for categories identified as male or female-skewed by each measure. Box plots show interquartile range (IQR) +/- 1.5 X IQR.

A.1.10 Robustness to Normalization Procedure

One advantage of the min-max normalization procedure we deploy is that it maintains the meaning of "0" in the gender dimension of the underlying word embedding model, such that all categories with gender associations above (below) 0 are male (female) skewed according to both the raw and normalized gender association. For robustness, Fig. S9 demonstrates that our results hold under a simpler alternative approach that min-max normalizes the raw, signed textual associations produced in the word embedding model, such that the category with the strongest negative association (-0.42, "chairwoman") is represented as -1 and the category with the strongest positive association (0.33, "guy") is represented as 1. A shortcoming of this approach is that, given the male-skew of the categories examined, the median point of this normalized distribution does not correspond to "0" in the unnormalized gender dimension in the embedding space; this means that categories identified as male-skewed according to this alternative normalization (i.e., those identified as having more male associations than the center of the normalized gender dimension) may be female-skewed according to the original embedding space (note, this problem is even greater for word embedding models with greater gender skew for the same categories, Fig. S5). Nevertheless, Fig. S6 shows that our main results do not change when comparing Google Images to the Google News corpus using this alternative normalization procedure: Google Images present significantly higher male-skew than Google texts ($p < 0.001$, Fig. S6*A*) and stronger gender associations overall for both male and female-skewed categories ($p < 0.001$ for both comparisons, Fig. S6*B*), (Wilcoxon signed-rank test, two-tailed).

A.1.11 Robustness to the Number of Categories Examined

Here, we illustrate the robustness of our results to the number of search terms examined. We focus on our main Google Image dataset collected without requesting gender-specific search results for each category (i.e. searching for "doctor", not "female doctor"). For this test, we randomly sample n search terms without replacement and calculate the average fraction of male faces observed across this subset of search terms. We repeat this procedure 50 times for each value of n search terms, such that the average fraction of male faces is measured for each of the 50 unique subsets of n search terms. Fig. S7 displays the proportion of male faces observed across each randomly sampled subset of n search terms. The results indicate that in each simulation, across each value of n search terms, the proportion of male faces observed is significantly above 50%, suggesting a consistent and stable bias toward male representation that holds independently of the number of search terms examined. This test

rules out the possible concern that our results are driven by a small subset of unusually biased social categories; instead, our analyses suggest that the male bias in Google Image search results generalizes robustly across categories.

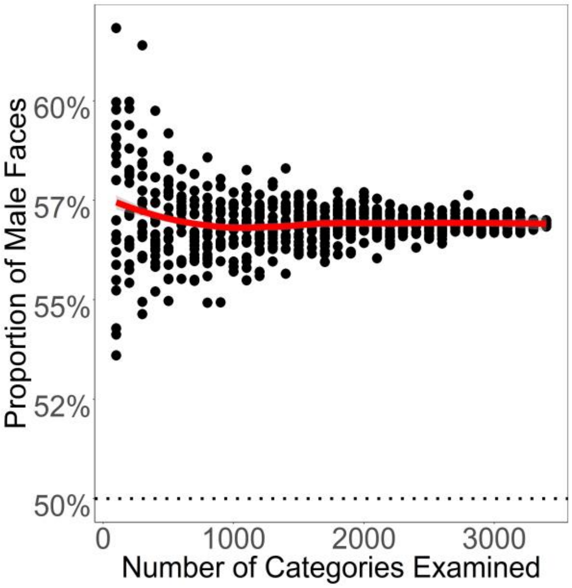

Fig. S7: The average proportion of male faces (y-axis) observed across 50 random samples (without replacement) of *n* search terms (x-axis). The proportion of male faces is calculated for each random sample of *n* search terms and is then averaged across all search terms within a given random sample. This analysis is based on our main Google Image dataset collected without requesting gender-specific search results for each category (i.e. searching for "doctor", not "female doctor"). The plotted line represents a loess smoothed regression (span = 0.75), aggregating over the mean values calculated across the 50 unique random samples of categories for each fixed number of categories (along the horizontal axis). The error band displays 95% confidence intervals of this fitted loess regression.

A.1.12 Replication using only Categories that have the Same Gender Association across All Modalities when Comparing Images and Text

Here, we test whether the main results in Fig. 1B hold while studying only those categories which have consistent female (male) associations across all modalities. We then compare the strength of gender associations between image and text, specifically for these consistently gender-skewed categories. We ensure that the specific categories in each condition remain constant throughout the analysis. Specifically, we compare them to the same robustly estimated female-skewed and male-skewed categories. To achieve this, we first identified all female (male) categories based on their associations in images, texts, and human judgments. We then replicate the analysis presented in Fig. 1B while focusing on these categories with robustly identified gender associations. We do not group categories based on census

associations in this analysis, first, because people's beliefs often do not track census representation, and second, because we are interested in people's beliefs about the gender of social categories broadly, not just occupations. This method yielded 1,472 social categories, comprising 1,281 consistently male-skewed categories and 191 consistently female-skewed categories. We note that this asymmetry is partly due to the general bias toward male over-representation across categories in images, text, and human judgments. Since we hold the female and male categories constant across these measures in this analysis, all statistical tests to follow assume comparisons that are paired at the category level.

Extended Data Fig. 5 shows the results of this analysis. We find that images present a significantly stronger level of gender bias for both female- and male-skewed categories. Specifically, when we compare the textual and image representations of these 191 strongly female-skewed categories, texts indicate an average gender association of -0.43 while images present a significantly higher gender association of -0.49, marking a significant 0.06 increase in absolute magnitude (p = 0.003, t-test, two-tailed, with paired comparisons at the category level). Similarly, when we contrast textual and image representations of the 1,281 strongly male-skewed categories, we find that texts indicate an average gender association of 0.23, whereas images indicate an average gender association of 0.46. This represents a two-fold increase in the absolute strength of male representation ($p < 0.001$, t-test, two-tailed, with paired comparisons at the category level). In this way, we find not only that images present a significantly stronger gender bias for both strongly female and male categories, but we also find that this effect of images is particularly strong in the context of amplifying male gender bias. This provides additional insight into the role that images play in amplifying male representation across categories.

A.1.13 Robustness to Machine Learning Classifications

In this section, we confirm that our results equally hold when using machine learning to classify the gender of the faces in our entire Google image dataset. We present these analyses in the supplementary material because automatically predicting the gender of faces in image data continues to present a difficult challenge for machine learning given the prevalence of demographic-related biases in the performance of such algorithms; e.g. prior work shows that popular machine learning algorithms are significantly better at classifying white male faces than any other demographic group [23,24]. We present these findings as one of several methods for evaluating whether our results are robust to controlling for subjectivity in human annotators. We note that machine learning cannot identify patterns that are entirely independent of cognitive biases in human annotators, since facial detection algorithms rely on annotated training data labeled by humans.

To automatically classify the gender of faces in our Google image dataset, we used the open-source facial detection algorithm provided by the OpenCV module in Python. We ran all of the raw Google images in our dataset through OpenCV's pre-trained facial classifier and gathered the gender classifications it associated with each face in each image. We applied this algorithm to both the raw, uncropped version of our image data, and to the dataset composed specifically of cropped faces in isolation. OpenCV was able to assign a gender classification to

417,882 faces in the uncropped dataset, and to 586,214 faces in the cropped dataset. We examine these automated gender classifications of both the cropped and uncropped versions of our dataset to provide another robustness test for demonstrating that our main results are not an artifact of the cropping algorithm applied in the pre-processing of our main dataset.

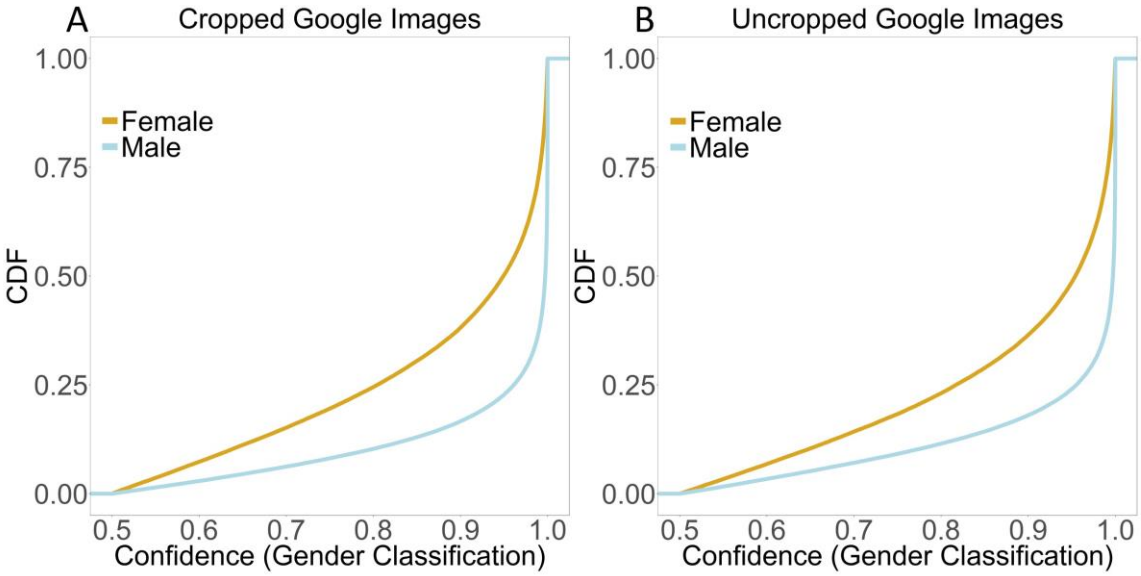

Fig. S8: The cumulative distribution function (CDF) of OpenCV's confidence (from 0.5 to 1) in its gender classifications of images in the (A) cropped and (B) uncropped version of our entire Google image dataset.

Fig. S8 shows that, consistent with prior studies on biases in deep learning facial classifiers, OpenCV was significantly less confident in its gender classifications when labeling faces as female rather than male, averaging 88.1% (88.6%) confidence for female faces in the cropped (uncropped) images, while averaging 94.6% (94.3%) confidence for male faces in the cropped (uncropped) images (Panel A displays the cropped and panel B displays the uncropped results; $p < 0.001$ for all comparisons, Wilcoxon rank sum test, two-tailed).

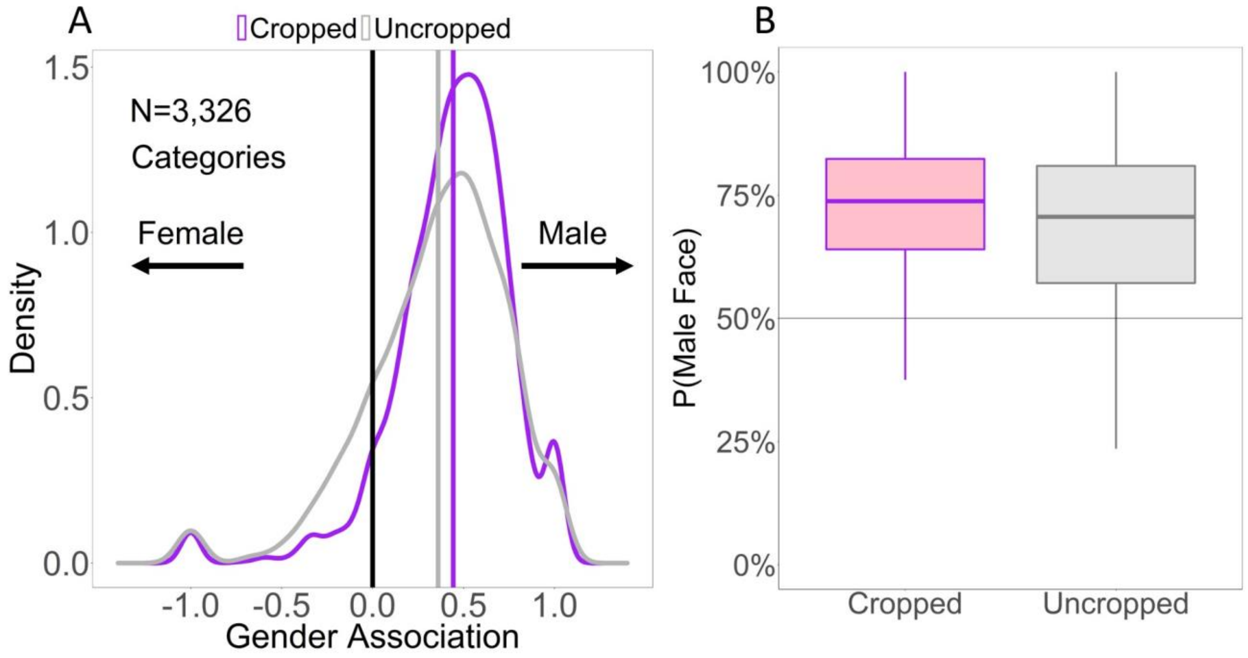

Fig. S9: (A) The distribution of gender associations for all categories in our dataset according to OpenCV's classifications. 3,326 categories could be associated with an overall gender association based on these classifications. (B) The probability of observing a face at the category level shown for all categories (n = 3,326) with images that OpenCV could successfully classify. The data shown reflect our main Google image dataset (i.e., the Google images collected when searching without specifying a specific gender in the search). Box plots show interquartile range (IQR) +/- 1.5 X IQR.

Fig. S9 further shows that our main results concerning the over-representation of males across social categories continues to hold when relying on deep learning classifications. The data shown in this analysis represents our main Google image dataset (i.e., the Google images collected when searching without specifying a gender in the search). 3,326 categories in this dataset could be associated with an overall gender association based on these machine learning classifications. Panel A of Fig. S9 shows that the overall gender association across these 3,326 categories is skewed male, averaging 0.44 and 0.35 along the gender scale for the cropped and uncropped datasets respectively, each of which is highly statistically significant at the $p < 0.001$ level (Wilcoxon signed-rank test, two-tailed). Ergo, the gender associations in the machine learning classifications of both the cropped and uncropped datasets are also significantly more male than the gender associations according to our main textual measures ($p < 0.001$ for all comparisons), while also being stronger overall in the magnitude of gender bias for both male and female categories compared to our measure of texts from Google News ($p < 0.001$ for all comparisons; Wilcoxon signed-rank test, two-tailed).

Panel B of Fig. S9 further shows that social categories are significantly more likely to be associated with male faces in both the cropped and uncropped datasets, according to machine learning. OpenCV identifies 73% of faces as male in the cropped dataset and 67% of

faces as male in the uncropped dataset (both are significantly higher than the 56% of male-skewed categories according to our main textual measure, and the 62% of male-skewed categories according to our image measure based on human annotators; $p < 0.001$ for all comparisons, proportion test, two-tailed).

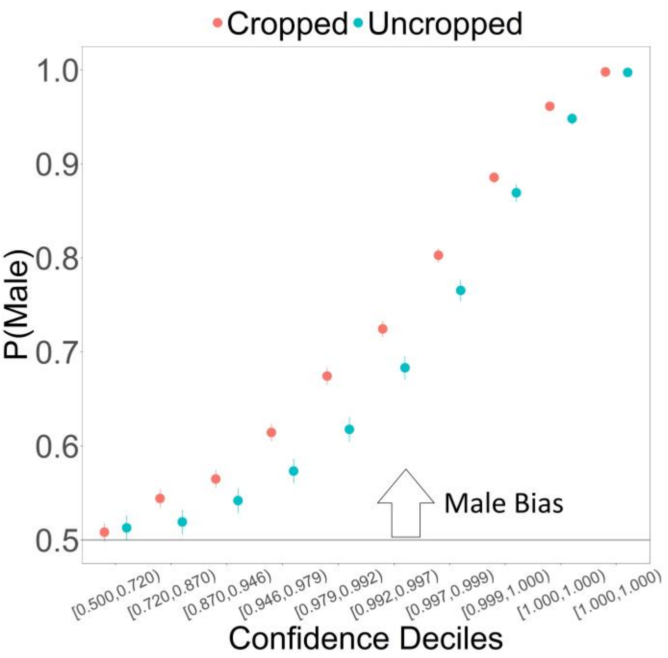

Fig. S10: The proportion of male faces (vertical axis) in all images within each decile of OpenCV's associated confidence levels (horizontal axis). For example, the data point within the third decile reflects the proportion of male faces across all images associated with a confidence in the range of 0.87 to 0.946. Error bars show 95% confidence intervals. The data in this analysis includes all Google images collected without searching specifically for gendered search results (e.g. excluding images associated by gendered searches such as "male doctor" and "female doctor").

Importantly, the bias toward over-representation of men according to machine learning gender classifications continues to hold when controlling for OpenCV's confidence levels in its classifications. Fig. S10 shows the proportion of male faces across all images that fall within each decile of OpenCV's associated confidence levels. For example, the data point within the third decile reflects the proportion of male faces across all images associated with an OpenCV confidence level in the range of 0.87 to 0.946. Fig. S10 shows that the bias toward the over-representation of males in our Google image data continues to significantly hold across all levels of confidence (at the $p < 0.01$ level or higher, proportion test, two-tailed), for both the cropped and uncropped image data sets.

These results concerning the over-representation of men in Google images is consistent with patterns of male over-representation observed in prior studies which used machine learning to classify the gender of faces in smaller, earlier samples of online images. For example, one of the most popular databases for training facial recognition algorithms is the Labeled Faces in the Wild (LFW) dataset [25], which consists of roughly 13k images assembled in 2007 from online news articles and image captions. For years, the composition of this dataset was not deeply studied, until Han & Jain (2014) [26] used machine learning to determine that 77% of the faces in the LFW were male. More recently, in 2015, the United States Office of the Director of National Intelligence released a face image dataset called IJB-A [27], described as a collection of roughly 25k images from across the web; in 2017, researchers showed that 75% of the faces in the IJB-A dataset were likely male [23]. While these datasets differ considerably from our own dataset in terms of their size, their data sources, their manner of curation, and the timing of their curation, they nevertheless exhibit a highly similar rate of male over-representation according to machine learning classifications of gender (73% male in our 2020 cropped Google dataset, 77% in the 2007 LFW dataset, and 75% in the 2015 IJB-A dataset). This indicates that the over-representation of men observed in our dataset is consistent with stable trends of male bias across a myriad of online sources and image datasets collected as early as 2007.

A.1.14 Robustness to Controlling for Linguistic Properties of Social Categories

In this section, we show that our main results hold when controlling for a range of linguistic features of social categories, namely: number of ngrams, category ambiguity, the frequency of a category in everyday language use, and whether categories contain explicit gender connotations.

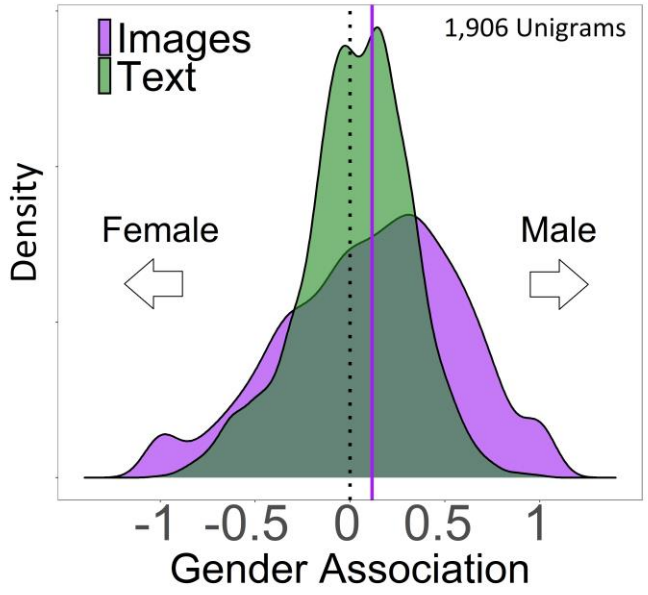

Fig. S11: The distribution of gender associations in images from Google Images and texts from Google News for 1,906 social categories (excluding all categories from our

main analysis that are not unigrams, e.g., "professional dancer"). The image-based measure captures the frequency of male and female faces associated with each category in Google Image search results (-1 means 100% female; 1 means 100% male); the text-based measure captures the frequency at which each category is associated with male or female terms in the Google News corpus of over 100 billion words (-1 means 100% female; 1 means 100% male). Solid green (purple) line indicates the average gender association according to text (images).

Fig. S11 shows that our main results hold when analyzing only social categories in Wordnet that consist of a single term (i.e., unigrams). Fig. S11 compares gender associations across images and text using only the 1,902 unigram categories in WordNet. The results show that the Google Images for these categories skew significantly toward male representation, both in general ($p < 0.0001$) and in comparison to text ($p < 0.0001$; Wilcoxon signed-rank test). In this way, we show that our results are not driven by idiosyncrasies introduced by bigram categories. Note, the same trend equally holds for bigram categories ($p < 0.0001$).

Next, we use a regression approach to evaluate our results while controlling for category polysemy, which refers to when categories have multiple definitions. The category *doctor*, for instance, can refer to someone who completed a doctoral graduate degree or more typically to a clinician, such that *doctor* is more polysemous than the category *plumber*, which likely only refers to one possible group. We measured polysemy using WordNet, which lists the conventional definitions associated with each category; to measure polysemy, we counted the number of unique definitions associated with each category in WordNet [28]. In the same model, we also control for the frequency of each word in terms of how commonly it is used in daily language. Word frequency was measured by matching each social category to the Exquisite Corpus by Luminoso (made available through the python package wordfreq), which identifies the frequency of English words and phrases across Google News, Google Books, Wikipedia, Twitter, and Reddit.

	**Strength of Gender Bias**		
Predictors	*Estimates*	*CI*	*p*
(Intercept)	0.39	0.38 – 0.40	**<0.001**
Measure [Text]	-0.17	-0.18 – -0.16	**<0.001**
Word.Frequency.Scaled	0.01	0.01 – 0.02	**0.007**
Polysemy	0.00	-0.00 – 0.00	0.611
Observations	6411		
R^2 / R^2 adjusted	0.124 / 0.124		

Table S4: An OLS regression predicting a category's overall strength of gender associations, as a function of (i) the data source (either images from Google Images or texts from Google News), (ii) the frequency of each category's use in online texts,

including Google News, Google Books, Twitter, and Reddit (scaled by standard deviation), and (iii) the polysemy of each category according to WordNet (where polysemy is measured as the number of different definitions associated with each category). Data are shown for all 3,495 categories. Standard errors are clustered at the category level. *CI*, 95% confidence intervals.

Table S4 presents the results of an OLS regression predicting a category's overall strength of gender associations (from 0 to 1), as a function of (i) the data source (either images from Google Images or texts from Google News), (ii) the frequency of each category's use in online texts, including Google News, Google Books, Twitter, and Reddit, and (iii) the polysemy of each category according to WordNet (where polysemy is measured as the number of different definitions associated with each category). Table S4 shows that textual measures of Google News are associated with significantly weaker gender associations than Google Images (β = -0.17, CI = [-0.18 - -0.16], p < 0.001), controlling for the polysemy and the frequency of each category in the English language. Additionally, table S4 shows that the polysemy of each category fails to predict the strength of gender associations in either data source (β < 0.01, p = 0.61); similarly, word frequency is weakly associated with the strength of gender association with each category (β = 0.01, p = 0.007), suggesting that more frequent categories are associated with slightly stronger gender biases, controlling for whether the measurement of bias is made via text or images.

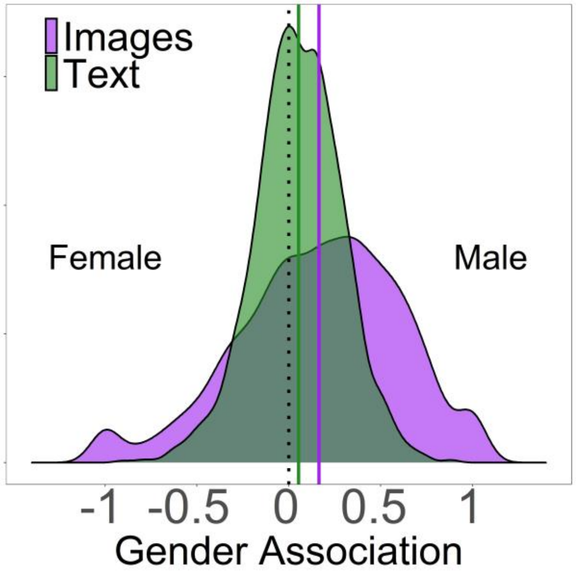

Fig. S12: The gender associations for all 2,922 non-gendered social categories in WordNet according to Google Images (purple) and textual embeddings of Google News (green). Solid purple (green) vertical lines indicate the average gender association according to images (text).

Finally, we show that our main results are highly robust to the exclusion of explicitly gendered categories, such as brother, sister, uncle, and aunt. There was no significant gender bias in the gendered categories that were excluded (52% were male). Excluding these categories left 2,922 non-gendered social categories in WordNet for analysis. Fig. S12 compares gender associations across images and text using only the 2,922 non-gendered categories in WordNet. We show that Google Images of non-gendered categories skew significantly toward male representation, both in general ($p < 0.0001$, average gender association 0.16) and in comparison to text ($p < 0.0001$, average gender association 0.05) (Wilcoxon signed-rank test, two-tailed). Similarly, when examining only non-gendered categories, the strength of gender associations remains significantly higher in images than text for categories with male-skewed ($p < 0.0001$) and female-skewed ($p < 0.0001$) associations (Wilcoxon signed-rank test, two-tailed). Thus, these results show that our main findings are robust to controlling for whether categories are explicitly linguistically gendered.

A.1.15 Robustness to Alternative Constructions of the Gender Dimension

Here, we test the robustness of our main results to different methods for defining the female and male centroids used to construct the gender dimension in embedding space. Our main analyses replicate the Kozlowksi et al. (2019) [19] method for defining gendered centroids using five gendered word pairs - that is, *woman*, *her*, *she*, *female*, and *girl* for the female centroid, and *man*, *him*, *he*, *male*, and *boy* for the male centroid. Here, we show that all of our main results equally hold while (i) using fewer gendered terms to define the gender centroids, and (ii) when using up to three times more gendered terms to define the centroids. Specifically, we compare the results from our main method (version 1) to three alternative versions of the gendered centroids:

> **Version 2**, where the female (male) centroid is defined only by woman (man), female (male), and girl (boy);
>
> **Version 3**, where the female (male) centroid is defined only by woman (man), her (him), she (he), female (male), girl (boy), feminine (masculine), and womanly (manly); and
>
> **Version 4**, where the female (male) centroid is defined only by woman (man), her (him), she (he), female (male), girl (boy), feminine (masculine), womanly (manly); mother (father), sister (brother), daughter (son), grandmother (grandfather), granddaughter (grandson), aunt (uncle), girlfriend (boyfriend), and wife (husband).

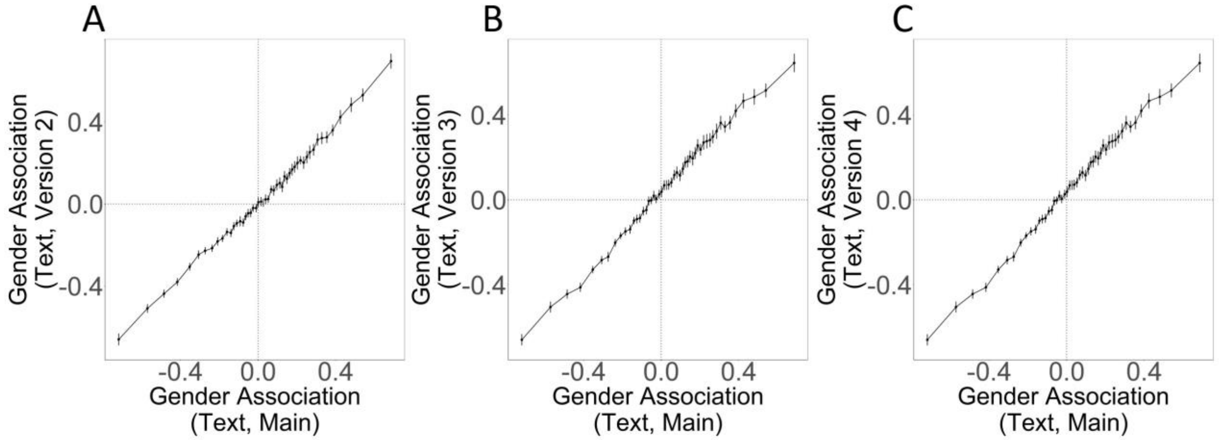

Fig. S13: The gender associations for all social categories in WordNet (n = 3,434) according to textual word2vec embeddings of Google News, under different representations of the gender dimension. Correlating the gender associations between the main gender dimension in our study and (A) version 2, (B) version 3, and (C) version 4 of the gender dimension. Data points show mean values across evenly spaced bins along the horizontal axis, and error bars display 95% confidence intervals.

Fig. S13 examines the correlation of the gender associations between the representation of the textual gender dimension used in our main study and three alternative versions of the textual gender dimension. All panels of Fig. S13 illustrate that, across all 3,434 social categories, the gender association of categories is highly correlated across these alternative gender dimensions (p < 0.0001 for all comparisons, Pearson Correlation, two-tailed), indicating that reasonable changes in the gender dimension have minimal impact on the gender associations of categories.

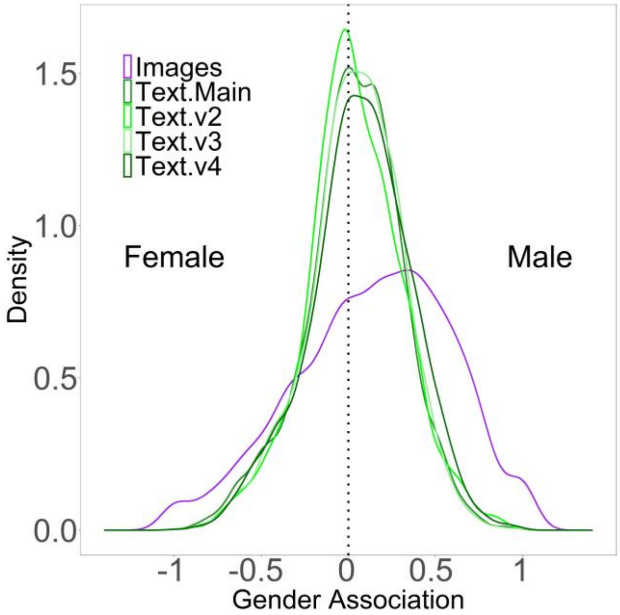

Fig. S14: The distribution of gender associations for 2,986 social categories in images from Google Images and texts from Google News, while varying the construction of the gender dimension used to score the gender association of categories in the word embedding model of Google News. The image-based measure captures the frequency of male and female faces associated with each category in Google Image search results (-1 means 100% female; 1 means 100% male); the text-based measures capture the frequency at which each category is associated with male or female terms in the Google News corpus of over 100 billion words (-1 means 100% female; 1 means 100% male associations).

Fig. S14 goes further by confirming that our main results comparing gender associations across images and text equally hold under alternative representations of the gender dimension. Men are significantly over-represented in Google Images compared to Google News, regardless of how the gender dimension is constructed ($p < 0.0001$ for all comparisons, Wilcoxon signed-rank test); and the overall magnitude of the gender associations, in either the male or female direction, are similarly stronger in Google Images as compared to Google News, regardless of how the gender dimension is constructed ($p < 0.0001$ for all comparisons, Wilcoxon signed-rank test, two-tailed).

Another potential concern is whether the gender dimension we rely on, which is centered on gender neutrality (at 0), is an appropriate method for evaluating biases in the amplification of gender associations, since a measure of amplification could in theory account for what we know about the existing empirical distribution of gender within a category. In other words, we could compare the gender associations produced by texts and images after centering them on the true gender distribution for each category according to empirical data such as the US census. This is the analysis we conduct below.

For this analysis, we begin by calculating the same gender dimension for the Census data (which contained 685 occupational categories that matched our text and image datasets). Then, for each category, we treat the census value along the gender dimension as "0" and then center the gender associations in text and image on this census value. So, for example, if the census data identifies *plumber* as a 0.4 along the gender dimension, we then set this value as the center point in the distribution against which we compare the position of *plumber* based on its gender association in image and text data.

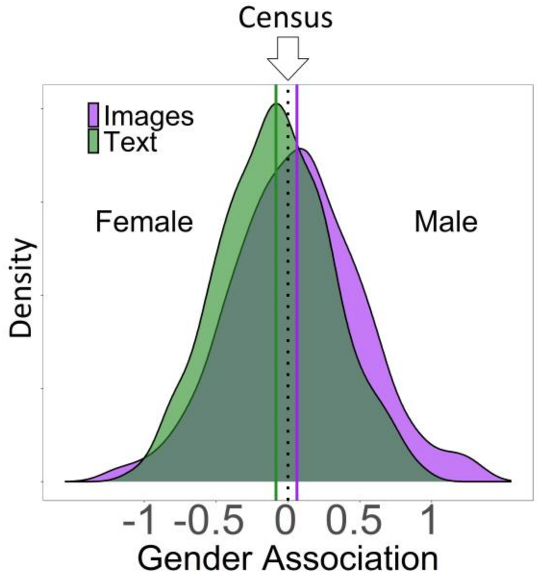

Fig. S15: The distribution of gender associations in images from Google Images and texts from Google News for 685 occupations. For each occupation, we set the value of 0 to the fraction of males that belong to each occupation, according to the US census bureau. To center the distributions accordingly, we first calculate the gender associations according to our standard measure for each data source; we apply the same procedure to the gender associations according to the census, and then for each category, we subtract the census' gender balance from each data source, thereby centering them on the census' representation. Positive values thereby indicate that a data source presents greater male representation for a given occupation than the census, and negative values present greater female representation. Solid green (purple) line indicates the average gender association according to text (images).

The results of this analysis are shown in Fig. S15. We find that the textual measures are significantly more female relative to the census (μ = -0.08, $p < 0.0001$), whereas the image measures are significantly more male relative to the census (μ = 0.05, $p < 0.0001$), (Wilcoxon signed-rank test, two-tailed). The census-centered text and image values are highly significantly different from each other ($p < 0.0001$, Wilcoxon signed-rank test, two-tailed).

A.1.16 Robustness to Search Frequency in Google Images

Here, we illustrate that our results are robust to controlling for the frequency at which each social category is searched using Google's Image search engine. It is important to show that our results are robust to controlling for the frequency of searches to rule out the possibility that our results are driven by rare or infrequently searched categories. If the protruded over-representation of male faces in Google Images applied only to the infrequently searched categories, this would raise the concern that the male bias observed is a result of algorithmic noise in retrieving images for infrequent searches, rather than as a result of systematic male over-representation.

To acquire this data, we used the Google Trend interface[1], which allowed us to determine the frequency at which specific categories are searched in Google Images, averaged across the entire United States from August 2020 to August 2021. Google's frequency results are normalized along a 0 to 100 scale.

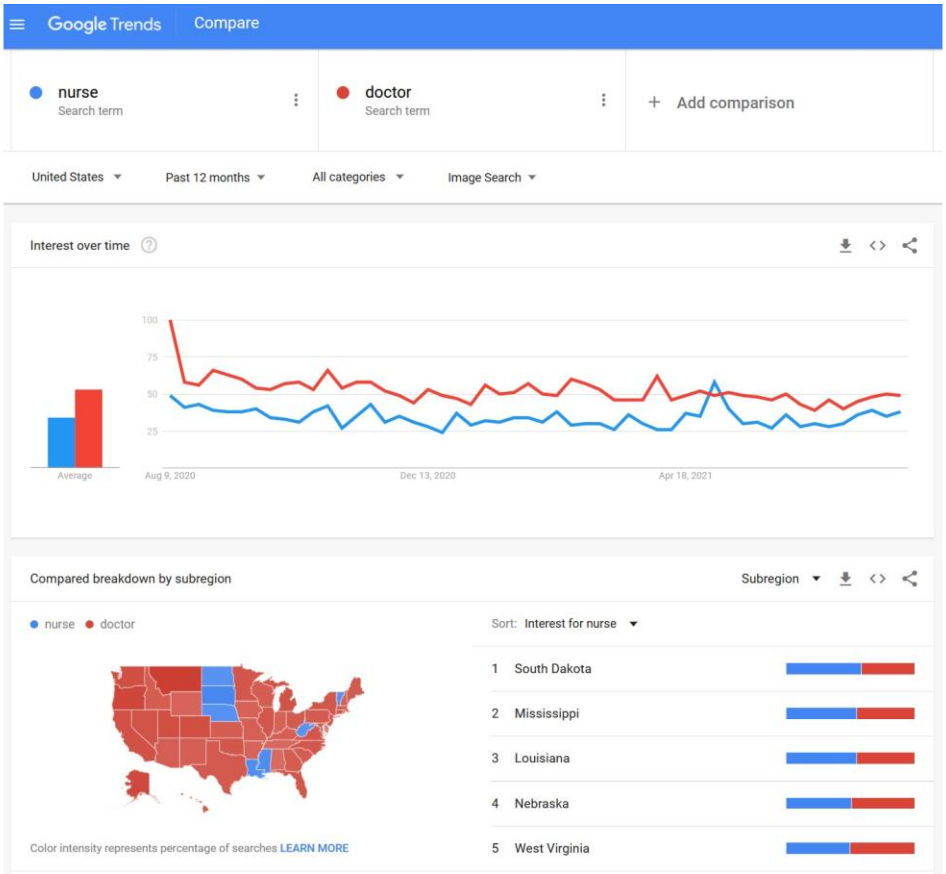

Fig. S16: A screenshot of the Google Trends interface used to gather data on the frequency at which each category in our dataset was searched via Google Images, averaged across the US from August 2020 to August 2021.

[1] https://trends.google.com/trends/?geo=US

Fig. S16 provides a screenshot of the Google Trend's interface and the information it provides. To ensure that we gathered properly standardized frequency data from the Google Trends API, every social category in our dataset (*n* = 3,495) was entered into the Google Trends API alongside the category *doctor*, since *doctor* was identified as an especially frequent category (see Fig. S16 for example). Only two categories — *doctor* and a different category — were searched at the same time. This method ensures that the search frequency results collected for each category are standardized to a common referent. This technique was implemented to control for the fact that the algorithm underlying Google's normalized measure of search frequency is not publicly available.

	**Proportion of Male Faces**			
Predictors	*Estimates*	*std. Error*	*CI*	*p*
(Intercept)	0.58	0.00	0.57 – 0.59	**<0.001**
Google.Img.US.Search.Freq	-0.10	0.04	-0.18 – -0.01	**0.026**
Observations	3410			
R^2 / R^2 adjusted	0.001 / 0.001			

Table S5: An OLS predicting the proportion of male faces associated with each social category as a function of the frequency at which each category is searched into Google Images, averaged across all searches in the US over the past year. Only faces that were identified as either male or female were included. *CI*, 95% confidence intervals.

Table S5 confirms that the male bias observed in our main analyses is not an artefact of search frequency. Search frequency is only weakly negatively correlated with the proportion of male faces across all categories (β = -0.1, p = 0.026, two-tailed). The baseline expectation for the representation of faces remains significantly and positively skewed toward male representation (58% of faces are expected to be male, Y = 0.58, p < 0.0001), controlling for the search frequency of each category.

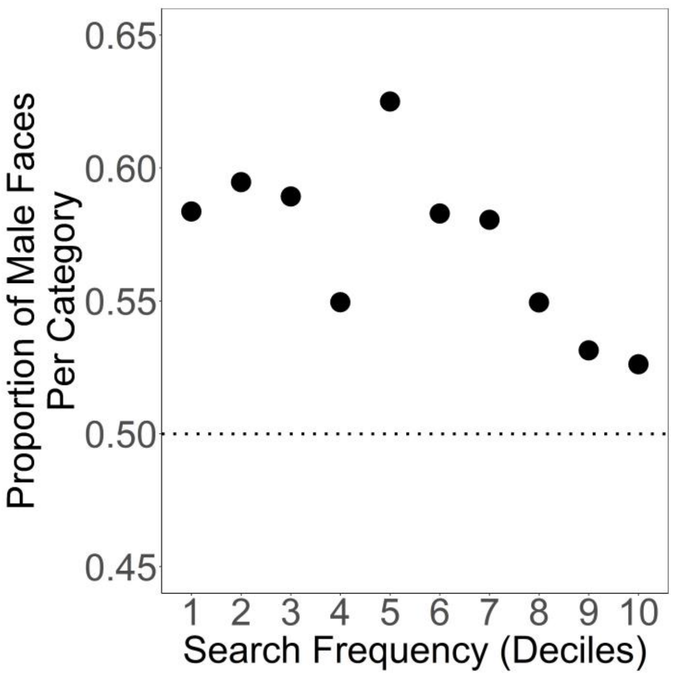

Fig. S17: The average proportion of male faces per category as a function of the frequency at which this category is searched in Google Images, averaged across all searches in the US over the past 12 months. Search frequency is grouped into deciles. Only faces that were identified as either male or female were included.

This result is confirmed visually by Fig. S17. Across all deciles of search frequency, the average proportion of male faces per category represents the majority (i.e., greater than 50% of faces).

A.1.17 Robustness to the Number and Ranking of Images in Google Image Search Results

Here, we evaluate the robustness of our results while controlling for heterogeneity across social categories in terms of the number of faces associated with each category in Google Image search results. On average, each social category was associated with 48 (σ = 44) faces in Google Images. To gain insight into what accounts for this heterogeneity, we find that the number of faces associated with each category in Google Images was positively and significantly predicted by the frequency with which the social category was searched in Google Images across the US ($r = 0.08$, $p < 0.0001$). In addition, the number of faces associated with each category in Google Images was also positively and significantly predicted by the frequency of each social category in the English language ($r = 0.13$, $p < 0.0001$, Pearson Correlation, two-tailed). These analyses suggest that the number of faces associated with a social category is non-randomly distributed across categories as a function of the overall frequency of categories in general language use and in Google search activity online.

	Strength of Gender Bias			
Predictors	*Estimates*	*std. Error*	*CI*	*p*
(Intercept)	0.22	0.00	0.21 – 0.23	**<0.001**
Measure [Images]	0.17	0.00	0.16 – 0.18	**<0.001**
Number of Faces	0.00	0.00	0.00 – 0.00	0.08
Google Img. US Search Freq	0.09	0.03	0.04 – 0.16	**0.003**
Word Frequency Scaled	0.00	0.00	0.00 – 0.02	0.07
Observations	6852			
R^2 / R^2 adjusted	0.125 / 0.125			

Table S6: An OLS predicting the absolute strength of gender bias for each category, according to either our main image (Google Images) or text (Google News) measure, as a function of the number of faces associated with each category, the frequency with which each category is searched across the US in Google Images, and the frequency with which each category is used in the English language. Standard errors are clustered at the category level. *CI*, 95% confidence intervals.

Importantly, Table S6 shows that the absolute strength of gender bias associated with each category is significantly higher in images than text ($\beta = 0.17$, $p < 0.001$), while controlling for the number of faces associated with each category, as well as the overall frequency of each category both in general language use and in Google Image search activity. Moreover, Table S6 shows that the number of faces associated with a category is statistically unrelated to the absolute strength of its associated gender bias ($\beta = 0.00$, $p = 0.08$), subject to these controls.

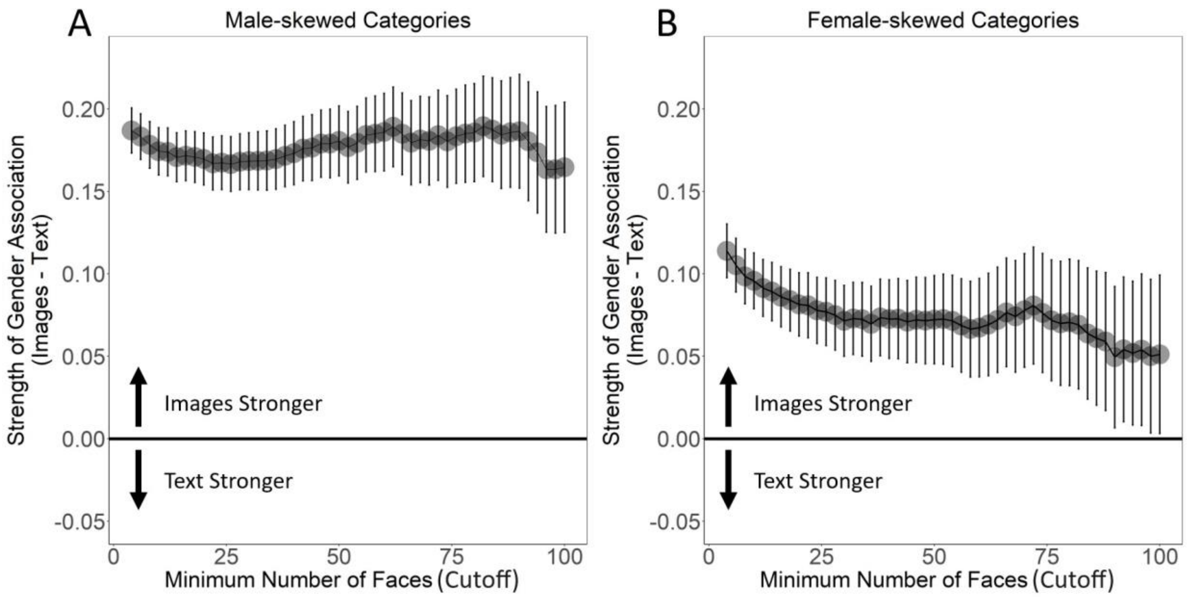

Fig. S18: The vertical axis shows the difference in the strength of gender bias associated with each category (n = 3,426) according to our image measure (Google Images) and text (Google News text) measure. This difference is displayed while only examining categories associated with a minimum number of faces in Google images; the minimum number of faces cutoff used to subset the data is shown on the horizontal axis (i.e., the vertical axis shows the results among only those categories that have at least the minimum number of faces shown along the horizontal axis). Results are shown separately for (A) male-skewed categories (n = 2,124) and (B) female-skewed categories (n = 1,181). In both panels, data points show mean values across evenly spaced bins along the horizontal axis, and error bars display 95% confidence intervals calculated using a t-test (two-tailed).

These results are corroborated by Fig. S18, which displays the difference in the strength of gender bias associated with each category according to our image and text measure, as a function of the number of faces associated with each category in Google images. For this analysis, we only examine categories associated with a minimum number of faces in Google Images. The minimum number of faces associated with each category is shown along the horizontal axis. For example, where the minimum number of faces is 25, this means that we subset our data to only compare categories across images and text where these categories are associated with at least 25 faces in Google Images. The results show that, across all cutoff points for the minimum number of faces considered, images continue to display significantly stronger gender bias than text at the $p < 0.05$ level for both male- and female-skewed categories. These results show that heterogeneity in the number of faces associated with social categories in Google Images is unlikely to serve as a confound driving our results.

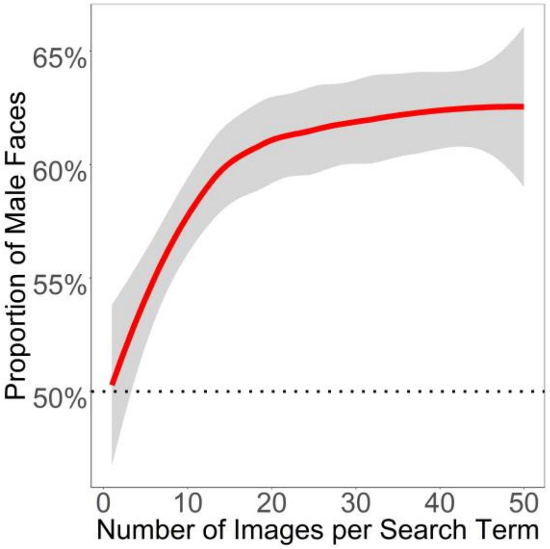

Fig. S19: The proportion of male faces observed, averaged across all search terms, when only examining a limited number of images for each search term. The number of images considered for each category is visualized along the horizontal axis. The vertical axis displays the expected proportion of male faces, averaged over 50 unique random samples of images (without replacement) for each search term, corresponding to each value along the horizontal axis. The plotted line represents a loess smoothed regression (span = 0.75), aggregating over the mean values calculated across the 50 unique random samples of images for each fixed number of images per search term (along the horizontal axis). The error band displays 95% confidence intervals of this fitted loess regression.

Next, we illustrate the robustness of our results to the ranking of images in Google's search results. Since Google's ranking of images may vary by the user doing the searching, we adopt a bootstrapping approach to simulate the effects of any possible ranking of the images on our outcome. For this test, we are primarily interested in whether examining only the top n images alters the proportion of male faces expected, while varying which images are included in the top n. For each value of n (from 1 to 50) — where n represents the number of top images examined — we randomly bootstrapped 20 possible sets of n images. For example, if n is 15, then we generated 20 unique subsets of 15 images (randomly sampled without replacement) for each social category, thus simulating 20 possible search result rankings constituting the top 15 images. Fig. S19 shows the expected proportion of male faces, averaged across all 20 unique subsets of each possible top n images. Across all possible top rankings of content, the majority of faces is expected to be male ($p < 0.0001$, two-tailed). Once the top 10 or more images are examined, the expected proportion of male faces converges to 60%, consistent with our main results identified using the top 100 images examined.

	Coefficients	CI
Number of Faces in Search Results	0.00<br>(0.00)	-0.00 - 0.00
Constant	0.57***<br>(0.00)	0.55 - 0.58
N	3,434	
R^2	0.00	

Standard errors in parentheses (clustered by coder)
* $p < 0.05$, ** $p < 0.01$, *** $p < 0.001$

Table S7: An OLS predicting the proportion of male faces associated with each social category as a function of the number of faces identified in each categories search results in Google Images. CI, 95% confidence intervals.

For additional robustness, we use an OLS to predict the proportion of male faces in a given category's Google Image search results as a function of the number of faces identified in the search results for each category. Table S7 shows that the probability of observing a male face in Google Image search results continues to be significantly higher (at 57%) than women (at 43%), holding constant the number of faces associated in Google Image search results with each category. In general, the number of faces in the top 100 Google Image search results associated with each category is not significantly predictive of the probability of observing male-biased search results (β = 0.00, p = 0.34). This robustness test indicates through yet another technique that our main results are unlikely to be confounded by differences among categories in their ability to return faces in their Google image search results.

A.1.18 Robustness to Evaluating Human Judgments of Uncropped Images

Here, we show that our results are robust to whether or not the faces in each image are automatically cropped prior to their classification by human coders. For this robustness test, we asked a separate sample of coders from Mechanical Turk to classify the gender of the focal faces in the uncropped Google images that we originally collected as part of our main study. Specifically, coders in this task were given two criteria when classifying the uncropped images: they were asked to focus on (i) the "focal face" of the person who (ii) belongs to the search category used to retrieve the image (e.g., "doctor"), following the standard methodology employed by recent crowdsourcing studies of gender and race bias in online images. For this task, we recruited a separate sample of 1,004 coders to classify the focal faces in all of the original, uncropped Google images associated with 300 categories randomly sampled from the broader set of 3,434 categories presented in the main text. Our pre-processing and data preparation procedures were otherwise identical to that of the main study; that is, we removed the judgments of all human coders who failed to complete a simple attention check (which was 13% of coders), and we excluded all images that were identified as failing to display a human face. This approach resulted in 21,146 images.

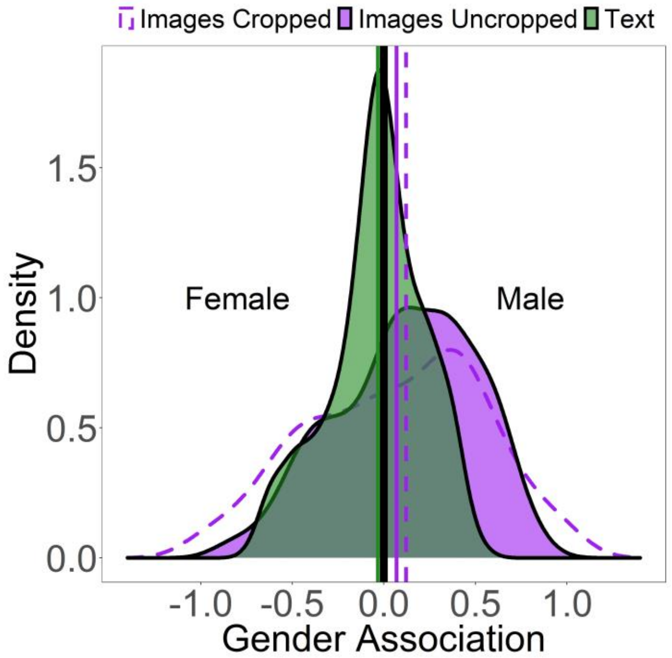

Fig. S20: The gender associations for 300 social categories in uncropped Google Images (Purple, Solid Line), cropped Google Images (Purple, Dashed Line), and Google News word embeddings (Green, Solid Line). Vertical lines indicate the average gender association according to each measure.

Within this uncropped sample, we find that the same patterns of gender bias hold with equal levels of statistical significance (Fig. S20). Indeed, 57% of images in the uncropped sample were identified as male, showing significant bias toward male representation ($p < 0.0001$, proportion test, two-tailed). Fig. S20 compares the gender associations according to Google News text embeddings with uncropped Google Images, across the same 300 categories. We find that uncropped Google images exhibited significantly stronger bias toward male representation than Google News text embeddings (t = 8.84, $p < 0.0001$, paired t-test, two-tailed), where these text embeddings exhibited a weakly significant bias toward female representation (t = -2.22, $p = 0.02$, t-test, two-tailed). Crucially, we show that the gender associations for each category were highly correlated between the uncropped and cropped image data ($r = 0.7$, CI = [0.64, 0.76], $p < 00001$); moreover, there is no significant difference in the gender associations between this cropped and uncropped sample of Google images (t = -0.64, $p = 0.49$, paired t-test, two-tailed). These results lend further support to our claim that our main results are unlikely to be driven by biases in our choice of cropping algorithm.

A.1.19 Validation of OpenCV Cropping Algorithm

A potential concern is the extent to which the OpenCV face-cropping algorithm used in this study is accurate and reliable, especially in light of prior work demonstrating demographic-related biases in popular face detection algorithms [23,24,29]. To ensure that our use of the OpenCV cropping algorithm did not introduce confounding variables due to gender-related biases in its cropping functionality, we examined the rate of false negatives (i.e., the rate at which the OpenCV algorithm missed faces in images) in a random sample of 1,000 randomly selected images from our dataset. We confirm that the rate at which the OpenCV algorithm missed faces in images (i.e., the false negative rate) was low (<8%), and manual inspection reveals that the majority of false negatives concern images where the faces are either too blurry or small to observe, or where the faces are occluded by headwear (e.g., helmets) or other objects, all of which are conditions where it is challenging for both humans and algorithms to identify faces, let alone their gender. For this same reason, these are the faces our hypotheses are least concerned with, since our goal is to characterize patterns of gender bias in those faces that are salient, interpretable, and most likely to be encountered by internet users. Most importantly, we observe that the false negative rate was equally conserved across both male and female faces. Among the faces that were missed and could be clearly identified, 48% were male and 52% were female, exhibiting no significant difference ($p = 0.27$, proportion test). This false negative rate is comparable to the performance of the OpenCV algorithm as measured in prior studies [30–32]. We also control for OpenCV's false positive rate, i.e., the rate at which it misidentified a non-face object as a human face. We asked each annotator to identify whether each image they classified displayed a human face. The annotators' judgments indicate that 18% of the cropped images were false positives and could not be reliably identified as a human face. As described in the main text, we control for false positives by removing all images for which at least one human coder identified the image as a false positive.

A.1.20 Controlling for the Number of Faces in each Image, the Number of Times each Image Repeats across Searches, and whether Images depict Avatars

Here, we examine the robustness of our main results to three important additional controls, namely: (*i*) the number of faces in each image, (*ii*) the number of times that each image repeats across searches, and (*iii*) whether or not the faces depicted are photographic or "avatars" (meaning cartoon or graphic illustrations).

We begin by reporting the relevant descriptive statistics. When examining our main Google Image dataset (collected without explicitly gendered searches), we find that 5% of images repeated within or across searches. We conducted this analysis by using image metadata comparisons to identify whether an image repeated across searches. This finding is re-assuring, since the uniqueness of most images in our data indicates that our findings are unlikely to be driven by images that repeat across multiple sources, such as stock photo images that are downloaded and reused across multiple sources. We add, however, that repeating images are still relevant to understanding which visual content is most available and widely circulated online in association with particular categories, and textual repetitions

in references to particular people or particular passages are also present in the training data on which the word embedding models we examine are trained.

In terms of avatars, we identified whether an image was an avatar using the same methodology for acquiring gender classifications: each of three unique coders was asked to identify whether the face was a cartoon avatar (yes or no), and we identified the modal judgment across coders to identify the final avatar classification for each image. Using this method, 16% of faces were identified as avatars. Table S8 shows that our main results significantly hold while controlling for these variables.

	Coefficients	Odds Ratio
Number of Faces in Image	0.00	1.00
	(0.00)	
Number of Image Repeats	-0.03	0.97
	(0.03)	
Cartoon/Avatar Face	-0.00	0.99
	(0.17)	
Constant	0.13***	1.15***
	(0.04)	
N	491,346	
R^2	0.00	

Standard errors in parentheses (clustered by coder)
* $p < 0.05$, ** $p < 0.01$, *** $p < 0.001$

Table S8: A logistic regression (binomial) predicting the probability of observing a male face, while controlling for (i) the number of faces associated with a given category in Google images, (ii) the number of times particular faces repeat within and across searches in Google images, and (iii) whether or not faces are identified as cartoon avatars. Standard errors are clustered at the level of social category (the results are the same if we cluster standard errors at the image level). The data examined in this model reflect our main Google image data collected without searching for explicitly gendered results (i.e., by collecting the top 100 Google images associated with single categories like "doctor" and "nurse"). ***, $p < 0.0001$.

Table S8 shows that male faces continue to be 1.15 times ($p < 0.001$) more likely to appear in Google images than female faces, even when controlling for the (i) the number of faces in each image, (ii) the number of times particular faces repeat within and across searches in Google images, and (iii) whether or not faces are identified as cartoon avatars. The number of faces associated with a given category in Google images was statistically unrelated to the probability of observing male faces (OR = 1.005). Similarly, the number of times an image repeated across searches was also not significantly correlated with the probability of observing a male face in Google image search results (OR = 0.97). Lastly, whether an image is a cartoon avatar or not bares no significant statistical relationship to the likelihood of observing a male face in our main image dataset (OR = 0.99).

A.1.21 Robustness to the number of images associated with each search

In Fig. S19, we showed that our main result concerning the over-representation of males continues to hold when examining randomly sampled subsets of decreasing size of the images returned with the search of each category. Here, we show that our results also hold when increasing the number of images associated with each search. In our main analyses, we collected the top 100 images for each category when searching with this category in Google's image search engine. Our choice to collect the top 100 images is consistent with the interface design of Google's image search engine, which provides approximately 100 images for each search, unless the user manually and explicitly requests for more images. Examining the top 100 images, therefore, is most consistent with examining the number of images that human users of Google are most likely to encounter. To confirm that our results are robust to the number of images collected for each category, we randomly selected 200 categories from our overall set of 3,495 categories, and for these categories we replicated our main data collection procedure while collecting up to 500 images from Google. When Google search results were unable to return 500 images for a given category, we also retrieved the recommended images associated with each of the images that were returned by the standard Google search. Using this method, we gathered 500 images for each social category. We then compared the proportion of male faces observed for each category when aggregating across the top 100 images and the top 500 images. We observe no significant difference in the proportion of male faces as a function of the number of images examined ($p = 0.43$, Wilcoxon signed-rank test, two-tailed).

A.1.22 Extended Analysis of Gender Associations in Images and Text as Compared to Census Data

In our main text, the results presented in Fig. 2C focus on the overall gender skew of occupations according to text and images. This comparison is made relative to the underlying empirical skew in the census data for the occupations available in our dataset. Fig. 2C shows that for the occupations in our dataset, the census data indicates a significantly overall male skew in the gender distribution of occupations. Yet, if we examine the skew of these occupations according to the image measure, we observe that the image representations of these same occupations are more biased toward male representation than the census data. Conversely, textual representations of the same occupations show no underlying gender bias, despite the clear male skew in the census data. An important question arises: are these results primarily a consequence of the over-representation of men in the image modality (consistent with our main argument)? Alternatively, could they be driven by the over-representation of women in the textual modality, or perhaps some combination of both factors? To address this question and offer further clarity on the statistical patterns underlying these findings, we build upon the analyses presented in our manuscript.

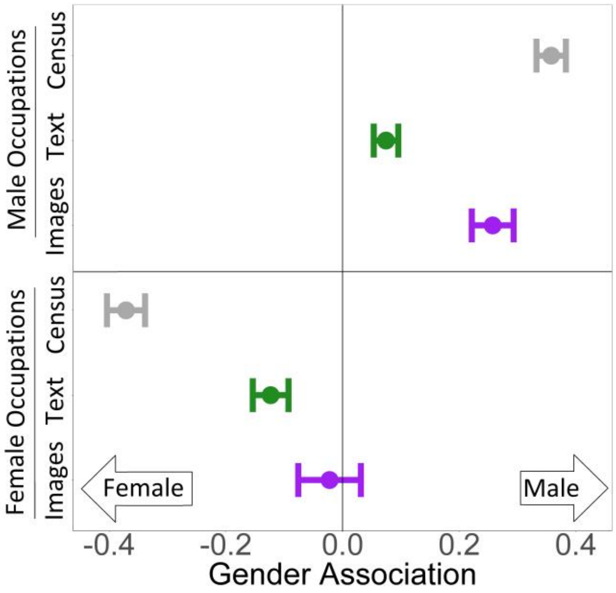

Fig. S21: The gender association of all matched occupations (n = 685) according to three sources (*i*) textual patterns in Google News (green), (*ii*) the empirical distribution of gender in the 2019 US census Bureau of Labor Statistics (grey), and (*iii*) Google Images (purple). These associations are categorized into whether a specific category is male-skewed or female-skewed according to the census data. Data are shown as mean values, and error bars display 95% confidence intervals.

Fig. S21 presents the same outcomes as Fig. 2C, while comparing the image, text, and census measures separately for occupations categorized as female-skewed or male-skewed occupations according to the census data. For both male- and female-skewed occupations in the census, the absolute strength of gender association is significantly stronger in the census compared to both the text and image modalities ($p < 0.001$, t-test, two-tailed). Yet, our main interest is in comparing the image and text measures to discern whether they are skewed toward female or male representation relative to each other and the census.

On the one hand, texts skew significantly more female for female-typed occupations compared to images ($p < 0.001$, t-test, two-tailed). Yet on the other hand, images skew significantly more male for male-typed occupations compared to texts ($p < 0.001$, t-test, two-tailed). The extent to which images are male-skewed for male-typed occupations is several fold greater than the extent to which texts are female-skewed for female-typed occupations ($p < 0.001$, t-test, two-tailed). In sum, images exhibit greater statistical bias overall, and in the direction of male over-representation. Indeed, we find that images exhibit significant male skew for male-skewed occupations in the census ($p < 0.001$), whereas images do not exhibit a significant female skew for female-skewed occupations in the census ($p = 0.41$), t-test, two-tailed). This finding indicates that the observed amplification toward male representation in the image modality compared to the census (as presented in Fig. 2C) is driven by the over-

representation of men in images depicting female-skewed occupations (according to the census), consistent with our main argument.

Relatedly, Fig. S21 shows that the gender skew of both female and male occupations in the census is significantly muted in the text modality, consistent with our theory. Crucially, we find that this muted effect is not biased in the text modality toward either male or female occupations; instead, across occupations, the aggregate gender skew according to the text modality is neutral, with no discernible bias toward either male or female representation. In this way, we provide statistical evidence for a bias toward the over-representation of men in the image modality relative to both ground truth census data and textual representations of occupations.

Supplementary Analyses (Experimental)

A.2.1 Robustness to Participants Searching in Google Instead of Google News

In our main experiment, participants in the text condition were guided to the Google News search bar and were asked to identify and upload a description of an occupation from this context. This design choice was made to maximize the similarity of the experimental context to our observational comparisons between Google Images and Google News. However, participants in our experiment (n = 150) were also randomized to a different version of the text condition, in which they were guided to the general Google search engine (i.e., simply google.com) and asked to perform the same task. Thus, in total, our experiment recruited 600 unique participants. 575 participants completed the task, exhibiting an attrition rate of 4.2%. We only examine data associated with participants who completed the task.

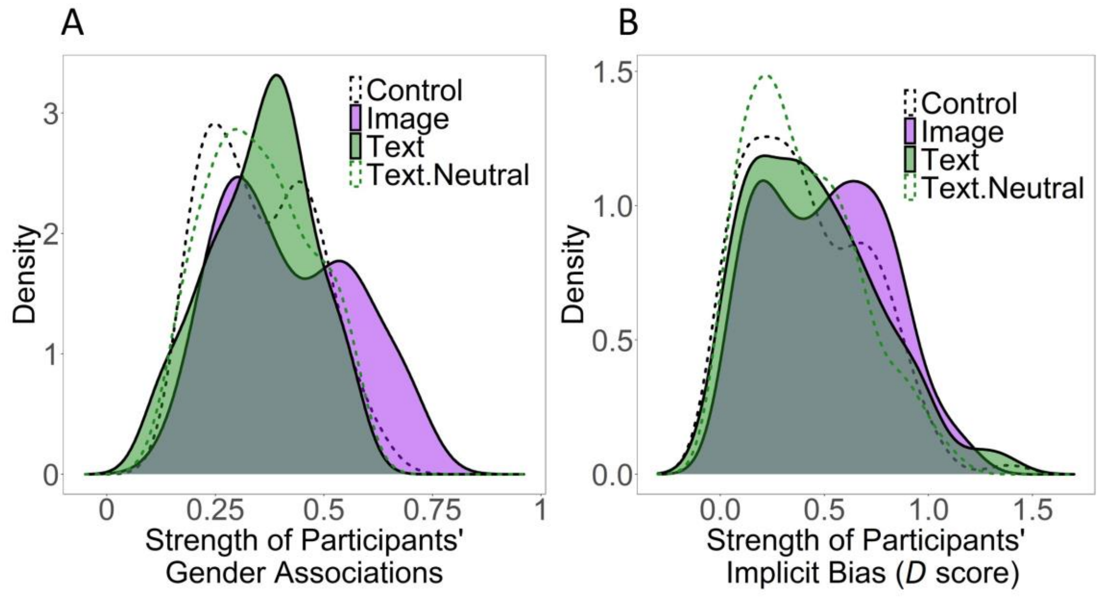

Fig. S22: Differential effects of googling for images rather than textual descriptions of occupations on participants' explicit and implicit gender biases, as compared to a

neutral control condition, while including a version of the text condition in which participants were directed to search for textual descriptions in the general Google search engine, rather than in Google News specifically (*n* = 575); the data shown for all other conditions are the same as those presented in our main results (see Fig. 3 in the main text). (A) The average absolute strength of the gender associations that participants reported for each occupation in each condition. (B) The average absolute strength of the implicit bias (*D* score) that participants exhibited in each condition. The results are shown in solid purple (image condition), solid green (Google News text condition), dotted black (control condition), and dotted green (General Google text condition).

Building on the presentation of our main experimental results, Fig. S22 compares the outcomes of each condition to an alternative version of the text condition in which participants were directed to retrieve textual descriptions of each occupation by searching via the general Google search engine (referred to as the "text neutral" condition) rather than Google News specifically. Panel A of Fig. S22 shows that there was no significant difference between the neutral text condition and the Google News text and control condition in terms of the strength of participants' self-reported gender associations for each occupation. Yet, again, we find that participants in the image condition produced significantly stronger gender associations compared to participants in the neutral text condition ($p < 0.001$, Wilcoxon signed-rank test, two-tailed), along with all other conditions (as discussed in the main text). Panel B of Fig. S22 shows that this result replicates when analyzing the overall strength of participants' implicit bias toward associated men with science and women with liberal arts; that is, we find no significant difference between participants' implicit bias in the text neutral and control condition, nor between the text neutral condition and main text condition; yet, participants in the image condition exhibited significantly stronger implicit biases than those in the text neutral condition ($p < 0.001$, Wilcoxon rank sum test, two-tailed). These results show that our main findings are not an artifact of comparing against Google News search results in particular, but instead generalize to a different means of acquiring textual descriptions of social categories via the Google search engine.

A.2.2 Robustness of Experimental Results to Controlling for Time and the Number of Sources

Here, we demonstrate that our experimental results are robust to controlling for how much time participants spent browsing and downloading descriptions (textual or visual) for each occupation, as well as controlling for the number of content sources they evaluated before selecting a description for each occupation. We measured the amount of time participants spent browsing, downloading, and uploading content for each occupation by using the Timer functionality in Qualtrics to track how long it took each participant to complete the task for each occupation presented (i.e., for each participant, we measured how long it took them to find content for each occupation they evaluated). We measured the number of sources that participants evaluated for each occupation by relying on their self-reported recollection; we

asked all participants in the Image condition to report how many images they looked at before making their final choice (for each occupation), and all participants in the Text condition to report on how many descriptions they looked at before making their final choice. This was asked for each occupation immediately after uploading the content for each occupation.

We begin by presenting the relevant descriptive statistics for our main Google News text condition and Google image condition. On average, participants in the Google News text condition spent 140 seconds (2.33 minutes) (min = 15.4 seconds; max = 1,740 seconds) browsing descriptions for each occupation (Panel A of Fig. S23), which was distributed across an average of 3.48 online sources (min= 1 source; max = 65 sources) (Panel B of Fig. S23), such that participants in this condition spent 55 seconds on average evaluating each potential online description. Participants in the Google image condition spent 81.6 seconds (1.35 minutes) (min = 9.48 seconds; max = 1,395 seconds) browsing images of each occupation (Panel A of Fig. S23), which was distributed across an average of 8.85 online sources (min = 1 source; max = 100 sources) (Panel B of Fig. S23), such that participants in this condition spent 21.8 seconds on average evaluating each potential online description. These descriptive statistics indicate that participants spent a considerable amount of time both browsing and evaluating online descriptions before making their content selections for each occupation, thus satisfying an important precondition for our theory, which assumes that people are paying sufficient attention to the descriptions they encounter online to permit these descriptions to prime (i.e., bias) their gender judgments.

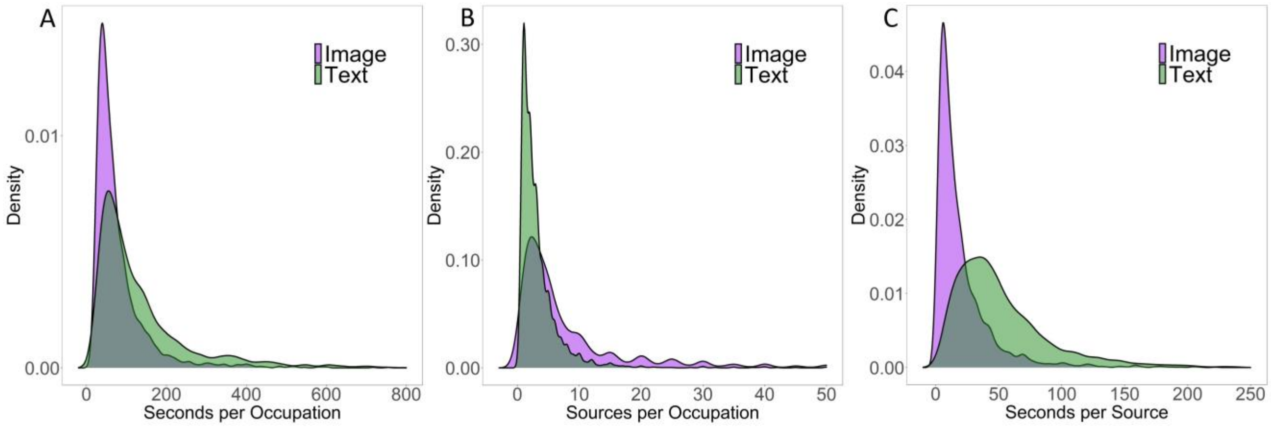

Fig. S23: (A) The number of seconds that each participant spent browsing and downloading descriptions for each occupation, split by the Google News text condition and the Google image condition; (B) the number of content sources each participant evaluated before selecting a description for each occupation split by the Google News text condition and the Google image condition; (C) The average number of seconds that each participant spent evaluating each online description before selecting a description for each occupation, split by the Google News text condition and the Google image condition.

Table S9 shows that participants in the text condition spent significantly more time browsing for online descriptions than those in the image condition ($p < 0.001$, t = 19.3, t-test, two-tailed), while participants in the image condition reported evaluating significantly more online sources than those in the text condition. These differences are likely due to the fact that, cognitively speaking, people tend to be faster at processing visual information than verbal information [33]. Importantly, however, we show that these differences do not confound our main experimental results. Table S9 presents the results of an OLS regression predicting the absolute strength of participants' gender associations as a function of experimental condition (the Google image vs. Google News text condition), while controlling for (i) the number of seconds participants spent browsing content for each occupation, (ii) the number of online sources participants encountered when identifying a description for each occupation, and (iii) the number of seconds that participants spent per online source for when seeking descriptions for each occupation. This model also includes fixed effects by occupation, as well as robust standard errors clustered at the participant level. We find that neither the time spent evaluating each occupation, nor the number of sources encountered for each occupation are significantly predictive of the absolute strength of participants' gender associations for each occupation. Crucially, we find that while holding time and the number of sources constant, participants in the image condition reported significantly stronger gender associations for each occupation than those in the text condition ($p < 0.01$, $\beta = 0.06$, CI = [0.05-0.08]).

	Coefficients	Confidence Interval
Experimental Condition [Image]	0.06** (0.02)	0.04 - 0.08
Seconds per Occupation	0.00 (0.00)	0.00 - 0.00
Sources per Occupation	-0.00 (0.00)	0.00 - 0.00
Seconds per Source	-0.00 (0.00)	0.00 - 0.00
Occupation Fixed Effects	Included	
Constant	0.42*** (0.03)	0.37 - 0.48
N	6527	
R^2	0.16	

Standard errors in parentheses (clustered by participant)
* $p < 0.05$, ** $p < 0.01$, *** $p < 0.001$

Table S9: An OLS regression predicting the absolute strength of participants' gender associations as a function of experimental condition (the Google Image vs. Google News text condition), while controlling for (i) the number of seconds participants spent browsing content for each occupation, (ii) the number of online sources participants encountered when identifying a description for each occupation, and (iii) the number of seconds that participants spent per online source for when seeking descriptions for each occupation. This model includes fixed effects for each

occupation, and its standard errors are clustered at the participant level. 95% confidence intervals are shown.

A.2.3 Robustness to Controlling for Participants' Gender

Here, we test the robustness of our experimental results when accounting for the self-reported gender of the participants completing the task. First, we examine whether participants are more likely to upload descriptions of occupations that correspond to their own gender (i.e., whether women are more likely to upload depictions of women in occupations, and similarly for men).

y = Gender of Uploaded Occupation Description	
Text Condition [vs. Image]	0.13***
	(0.02)
Participant Gender [Male]	0.03
	(0.02)
Occupation Fixed Effects	X
Constant	0.19*
	(0.07)
N	6527
R^2	0.20

Standard errors in parentheses (clustered by participant)
* $p < 0.05$, ** $p < 0.01$, *** $p < 0.001$

Table S10: An OLS regression predicting the gender of participants' uploaded descriptions of occupations (-1 = "female", 0 = no face shown or undecipherable face, 1 = "male"), as a function of participants' gender and experimental condition, with fixed effects by occupation. The control condition is excluded from this model because none of the content uploaded in the control condition was gendered (e.g., *apple*). Standard errors are clustered at the participant level and shown in parentheses.

Table S10 presents a model testing whether participants' gender significantly predicts the gender of the descriptions they upload across different occupations. The results show that there is no significant correlation between participants' gender and the gender of the descriptions they upload (β[Male] = 0.03, SE = 0.02, p = 0.12). Qualitatively, in the image condition, 56% of descriptions uploaded by female participants depicted women, and 53% of descriptions uploaded by male participants depicted men, illustrating stable trends across participant gender (none of the participants identified as non-binary in their gender). The same pattern holds in the text condition, albeit it had fewer gendered descriptions overall. In the text condition, both female and male participants uploaded nearly identical fractions of

male and female content: 12% (10.9%) of textual descriptions uploaded by women were female (male), and 11.9% (11.4%) of textual descriptions uploaded by men were female (male). These results collectively suggest that the experimental outcomes observed are not driven by underlying biases among participants toward uploading content that mirrors their own gender.

We further evaluate the robustness of our experimental outcomes to controlling for participant gender using two complementary statistical models. First, we test whether our main outcome of interest holds – namely, that the strength of participants' gender associations for occupations is significantly higher in the image condition – while controlling for the gender of the participant (self-identified as "male" or "female"; none of the participants identified as non-binary). Second, we test whether the same outcome holds when controlling for whether the self-identified gender of the participant matches the gender of the description they uploaded for a given occupation. This second analysis directly controls for any psychological biases participants may have toward exaggerating or reducing gender associations as a function of sharing the perceived gender of the target occupation.

	y = Strength of Gender Associations in Participant Responses		
	(1)	(2)	(3)
Image Condition [vs. Control]	0.06**	0.06**	0.06**
	(0.02)	(0.02)	(0.02)
Text Condition [vs. Control]	0.00	0.00	0.00
	(0.02)	(0.02)	(0.02)
Participant Gender [Male]		-0.03	-0.03*
		(0.02)	(0.02)
Participant Gender Match Upload Gender			0.15***
			(0.01)
Occupation Fixed Effects	X	X	X
Constant	0.43***	0.45***	0.39***
	(0.03)	(0.03)	(0.03)
N	9167	9167	9167
R^2	0.15	0.16	0.21

Standard errors in parentheses (clustered by participant)
* $p < 0.05$, ** $p < 0.01$, *** $p < 0.001$

Table S11: Models present OLS regressions predicting the absolute strength of participants' gender associations for occupations, corresponding to the experimental outcome presented in Fig. 3D in the main text. All models include standard errors clustered at the participant level.

Table S11 presents the aforementioned models. Model 1 shows the main experimental effect without controlling for the demographic of participants. Here, we see that being

randomly assigned to the image condition is associated with a significant increase in the average strength of participants' gender associations, while controlling for the occupation being evaluated and clustering standard errors at the participant level (β = 0.06, SE = 0.02, p < 0.01).

Model 2 presents the same model at Model 1 while controlling for the gender of the participants in our experiment. Model 2 finds no significant correlation between the gender of the participant and the absolute strength of participants gender associations for occupations (β[Male] = -0.03, SE = 0.02, p = 0.13); adding this demographic control variable has no impact on the main statistical effect of being randomly assigned to the image condition.

Model 3 replicates Model 2 while also including a dummy variable which captures whether a participant's gender matches the gender of the description they uploaded for a given occupation. Adding this variable significantly improves the R^2 of the model, an increase from 0.15 (Model 1) and 0.16 (Model 2) to 0.21 (Model 3) (p < 0.05, ANOVA). When a participant's self-identified gender matched the perceived gender of the content they uploaded for a given occupation, this was associated with a significant and sizable increase in the absolute strength of their gender association for this occupation (β = 0.15, SE = 0.01, p < 0.001). Importantly, including this variable again had no statistical impact on the strength or significance of the main experimental effect of being randomized to the image condition. That is, controlling for whether participants' self-identified gender matched the gender of their uploaded content, we continue to find that being randomized to the image condition was associated with a significant increase in the absolute strength of participants' gender associations relative to those randomized to the text and control condition (β = 0.06, SE = 0.02, p < 0.01; effect strength is identical to Model 1 and 2). In none of the models do we find that being randomly assigned to the text condition leads participants to exhibit statistically different gender associations for occupations compared to those randomized to the control condition. From these analyses, we conclude that our main experimental outcome of interest is robust to controlling for the gender of our experimental participants.

A.2.4 Using our Observational Measures of Gender Bias to Predict Gender Bias in Participants' Uploaded Descriptions and Self-report Beliefs

Here we confirm that our observational measures of gender bias in images from Google Images and texts from Google News are predictive of gender bias in the image/textual descriptions that participants uploaded in our experiment, as well as in their self-reported gender associations with each occupation. This analysis is important not only for demonstrating the ability for our observational measures to predict ecologically valid patterns of content exposure and response bias among human participants using Google, but also to confirm the correspondence between our alternative methods of measuring gender bias in text, namely the word embedding technique and the coarser method of counting the frequency of gendered descriptions in our experimental data.

The results of this analysis are presented in Extended Data Fig. 9. Panel A of Extended Data Fig. 9 shows that the distribution of gender in our observational sample of the top 100

Google images per occupation is highly correlated with the distribution of gender in the images uploaded by participants in our experiment (r = 0.74, p = 1.15 X 10^{-10}, n = 54 occupations, Pearson correlation, two-tailed). Similarly, panel B of Extended Data Fig. 9shows that the distribution of gender in our observational sample of the top 100 Google images per occupation is also highly correlated with participants' self-reported gender associations for each occupation, which they provided after uploading an image from Google (r = 0.76, p = 1.49 X 10^{-11}, n = 54 occupations, Pearson correlation, two-tailed). The same results hold for the text condition. Panel C of Extended Data Fig. 9 shows that the distribution of gender in our observational word embedding measures of Google News is highly correlated with the distribution of gender in the textual descriptions uploaded by participants in our experiment (r = 0.54, p = 2.88 X 10^{-5}, n = 54 occupations, Pearson correlation, two-tailed). This finding is particularly striking not only because gender bias is measured differently by our word embedding model and experimental analysis, but also because the Google News data underlying the word embedding model was collected in 2013, whereas our experimental data involves descriptions from Google News downloaded in 2022. Lastly, panel D of Extended Data Fig. 9shows that the distribution of gender in our observational word embedding measures of Google News is highly correlated with participants' self-reported gender associations for each occupation, which they provided after uploading a textual description from Google News (r = 0.8, p = 5.3 X 10^{-13}, n = 54 occupations, Pearson correlation, two-tailed).

	Fig. 3C y = Gender Association in Participants' Responses			Fig. 3D y = Strength of Gender Associations in Participant Responses		
	(1)	(2)	(3)	(4)	(5)	(6)
Image Condition	-0.05**	0.03	0.03	0.06***	-0.09***	-0.12***
	(0.01)	(0.04)	(0.04)	(0.01)	(0.02)	(0.03)
Gender Associations in Google Descriptions		0.66***	1.00***			
		(0.03)	(0.21)			
Gender Associations in Google Descriptions x Image Condition			-0.36			
			(0.21)			
Strength of Gender Associations in Google Descriptions					0.35***	0.20
					(0.03)	(0.14)
Strength of Gender Associations in Google Descriptions x Image Condition						0.17
						(0.14)
Constant	0.03	0.04	0.04	0.36***	0.31***	0.33***
	(0.04)	(0.03)	(0.04)	(0.01)	(0.01)	(0.02)
N	108	108	108	108	108	108
R^2	0.00	0.62	0.63	0.04	0.35	0.35

Standard errors in parentheses (clustered by occupation)
* $p < 0.05$, ** $p < 0.01$, *** $p < 0.001$

Table S12: Models 1-3 present OLS regressions predicting participants' gender associations for occupations, corresponding to the results presented in Fig. 3C in the main text; Models 4-6 present OLS regressions predicting the absolute strength of participants' gender associations for occupations, corresponding to the results

presented in Fig. 3D in the main text. All models include standard errors that are clustered at the occupation level in parentheses.

A.2.5 Robustness to Controlling for Experimental Condition (Fig. 3CD)

Here, we confirm that the correlations presented in Fig. 3C and Fig. 3D in the main text are robust to controlling for experimental condition, while clustering standard errors by occupation. Table S12 presents a series of models exploring these robustness tests. Models 1-3 present OLS regressions predicting participants' gender associations for occupations, corresponding to the results presented in Fig. 3C in the main text. Model 1 shows that participants randomized to the image condition exhibited significantly more female associations; however, this effect of experimental condition goes away once we control for the gender associations in the Google descriptions uploaded for each occupation in each condition (Models 1 and 2). Consistent with the results presented in Fig. 3C, Model 2 shows that the gender associations in the Google descriptions uploaded for each occupation in each condition is highly predictive of participants' self-reported gender associations, even when holding experimental condition constant (β = 0.66, p < 0.001). Model 3 confirms that the interaction is insignificant between experimental condition and the gender associations in the Google descriptions uploaded for each occupation. This is consistent with the claim that gender associations in the Google descriptions uploaded for each occupation predicts participants' self-reported gender associations across both experimental conditions.

Model 4 shows that participants randomized to the image condition exhibited significantly stronger gender associations overall (β = 0.06, p < 0.001). Consistent with the results presented in Fig. 3D, Model 5 shows that the strength of gender associations in the Google descriptions uploaded for each occupation in each condition is highly predictive of the strength of participants' self-reported gender associations, even when holding experimental condition constant (β = 0.35, p < 0.001). Model 6 confirms that the interaction is insignificant between experimental condition and the strength of gender associations in the Google descriptions uploaded for each occupation. This indicates that the strength of gender associations in the Google descriptions uploaded for each occupation predicts the strength of participants' self-reported gender associations across both experimental conditions. This supports our hypothesized mechanism, since the descriptions participants uploaded in the image condition exhibited significantly stronger gender bias than those uploaded in the text condition (Fig. 3A), which is highly predictive of an increase of gender bias in participants' self-reported gender associations.

A.2.6 Comparing the Strength of Gender Priming between Text and Images

In our main text, we focus on comparing the image and text condition in terms of the overall prevalence of gender biases in the descriptions that participants uploaded and the associated effects this has on the prevalence of gender biases in participants' explicit and implicit beliefs. Here, we evaluate the additional prediction that images are stronger at priming gender biases in people's beliefs, even when the images and texts being compared are explicitly gendered. Extended Data Fig. 10 shows that when comparing only participants who provided

explicitly gendered descriptions in the image and text condition, participants in the image condition reported significantly stronger gender biases in their associations with occupations (p = 5.09 X 10^{-6}, t = 4.58, MD = 0.06, t-test, two-sample, two-tailed), even when using a linear regression to control for the specific gender and the specific occupation associated with the uploaded description (β = 0.05, SE = 0.01, p = 2.08 X 10^{-5}). These findings indicate that even when gender is salient in both text and image, exposure to images leads to stronger biases in people's beliefs.

A limitation of the analysis strategy above is that it is unable to distinguish the priming effect of the specific descriptions uploaded by participants from the priming effect of the content that participants were exposed to prior to selecting content to upload. To address this limitation, we conduct an additional analysis which tests whether the main hypothesized priming effect of images - namely, that gendered images more strongly prime gendered responses than gendered textual descriptions - holds when controlling for the level of gender bias associated with each occupation in our observational sample (i.e., within our main sample of Google images and within our main word embedding measure of gender association in Google News). In this way, we leverage our observational sample as an estimate of the level of gender bias in the distribution of content that participants were exposed to when searching for descriptions of each occupation (see Extended Data Fig. 9 for statistical analyses showing that gender associations in our observational sample of images and text correlates effectively with the gender distribution of content uploaded by participants in the experiment). The results of this analysis are presented in Table S13.

	y = Strength of Participants' Gender Associations
Image Condition [vs. Text]	0.11***
	(0.01)
Strength of Gender Association in Observational Dataset	-0.06
	(0.04)
Occupation Fixed Effects	X
Participant Fixed Effects	X
Constant	0.31***
	(0.02)
N	3050
R^2	0.44

Standard errors in parentheses
* $p < 0.05$, ** $p < 0.01$, *** $p < 0.001$

Table S13: An OLS regression predicting the absolute strength of participants' gender associations for the occupations in our experiment, while controlling for experimental condition (the main treatment effect) and the level of gender bias

associated with each occupation in our observational sample (i.e., within our main sample of Google images and within our word embedding measures of gender association in Google News). This model includes fixed effects by occupation and participant. Standard errors are shown in parentheses. Data from the control condition is excluded from this analysis because in this condition the connection is severed between the observational sample and the occupations for which participants indicated their associated gender.

The model in table S13 predicts participants' absolute strength of gender associations, as a function of experimental condition, controlling for the absolute strength of gender bias associated with each occupation in our observational sample. This model also includes fixed effects by occupation and clusters standard errors at the participant level. Participant data from the control condition is excluded from this model because, in this condition, the connection is severed between the content participants encountered online and the occupations for which participants indicated their associated gender. We find that, even when controlling for the level of bias in the content distribution in images and text, being randomized to encounter and upload image content as opposed to textual content leads to a significant increase in the absolute strength of participants' gender associations (β[Image] = 0.11, SE = 0.01, $p < 0.001$). This analysis provides further evidence that, holding constant the expected level of gender bias in the content distribution participants encountered, exposure to images rather than textual descriptions of occupations leads to significantly stronger gender associations. This finding is compatible with a priming mechanism.

A.2.7 Robustness to Statistical Controls (Fig. 3E)

Here, we confirm that the correlation presented in Fig. 3E in the main text is robust to controlling for experimental condition and occupation fixed effects, while also clustering standard errors at the participant level (Table S14). We see that, subject to these controls, the absolute strength of participants' gender ratings continues to be positively and significantly correlated with their D score, which captures the extent to which they were biased toward associated men with science and women with liberal arts in the implicit association test administered immediately after the experiment ($\beta = 0.08$, $p = 0.02$). This correlation is significantly higher if we do not control for experimental condition ($\beta = 0.10$, $p = 0.007$) or if we exclude data from the control condition, while still controlling for experimental condition ($\beta = 0.13$, $p = 0.001$).

	Participants' *D* Score (Implicit Bias)			
**Predictors**	**Estimates**	**SE**	**Z**	***P***
(Intercept)	0.361	0.042	8.593	0.000
Strength of Explicit Gender Bias	0.088	0.037	2.352	0.019
Condition[Image]	0.105	0.044	2.417	0.016
Condition[Text]	0.060	0.045	1.345	0.179
Occupation Fixed Effects	Included			
Observations	8780			
R^2 / R^2 adjusted	0.026 / 0.02			

Table S14: An OLS regression predicting participants' *D* score (implicit bias) as measured by the IAT immediately after the experimental task as a function of the strength of participants' explicit gender ratings during the experiment, with fixed effects controlling for (i) experimental condition and (ii) occupation, while also clustering standard errors at the participant level.

A.2.8 Experimental Effects of Images on Implicit Bias Over Time

An important question that arises concerning our experimental results is whether the effect of googling for images, rather than texts, shapes people's gender associations in merely an ephemeral manner – akin to generic priming effects – or whether the effect we report has lingering consequences on people's implicit gender bias. To evaluate this possibility, we recruited the same participants who completed the first experiment to complete the same IAT three days after the experiment (91% of participants successfully returned to complete the IAT three days after, marking an attrition rate of 9%). Using this data, we tested the prediction that participants who were exposed to increasingly biased Google images during the first experiment will continue to report significantly stronger implicit bias toward associated men with science and women with liberal arts three days after the experiment.

	Coefficients	Confident Intervals
Day	-0.01<br>(0.01)	-0.03 - 0.01
Image Condition [vs. Control]	0.08*<br>(0.03)	0.01 - 0.14
Text Condition [vs. Control]	0.04<br>(0.04)	-0.02 - 0.11
Constant	0.38***<br>(0.03)	0.31 - 0.45
N	1612	
R^2	0.00	

Standard errors in parentheses (clustered by participant)
* $p < 0.05$, ** $p < 0.01$, *** $p < 0.001$

Table S15: An OLS predicting participants' implicit bias (as measured by the IAT's *D* score), as a function of the day in which they completed the IAT (Day = 0 or Day = 3), as well as the experimental condition to which they were assigned (the Google News text, Google image, or control condition). Standard errors are clustered at the participant level and shown in parentheses.

Table S15 shows the results of an OLS regression predicting participants' implicit bias (as measured by the IAT's *D* score), as a function of the day in which they completed the IAT (Day = 0 or Day = 3), as well as the experimental condition to which they were assigned (the Google News text, Google image, or control condition). Table S15 shows that while participants' implicit bias nominally decreased after three days (β = -0.01), this decrease was not statistically significant (p = 0.35, t = -0.92). Indeed, participants' implicit bias immediately after the experiment was highly correlated with their implicit bias rating three days after the experiment (r = 0.43, p < 0.001). Accordingly, table S15 shows that, even after three days, participants randomized to the image condition continue to exhibit significantly stronger implicit bias than participants randomized to the control condition (p < 0.05, β = 0.08, CI = [0.01-0.14]). However, after three days, there continued to be no significant difference in the level of implicit bias exhibited by participants randomized to the text condition and those randomized to the control condition (p = 0.18, β = 0.04, CI = [0.02-0.11]). We thus find evidence that the priming effects of Google images on participants' implicit biases lingered for several days after the initial experiment.

This finding is corroborated by an OLS model which predicts participants' implicit bias as a function of the strength of their explicit gender associations with each category as recorded during the initial experiment, while controlling for the day on which their implicit bias was measured (Day = 0 or Day = 3, relative to the experiment). We find that, for participants in the image condition, those who exhibited stronger explicit biases during the initial experiment continued to exhibit stronger implicit bias three days after the experiment (p < 0.01, β = 0.37, CI = [0.14-0.59]). In our main analyses, we show that participants randomized

to the image condition exhibited significantly stronger gender associations by occupation than those randomized to the Google News text condition (see Fig. 3). Together, these results indicate not only that exposure to online images led to stronger explicit and implicit gender biases, but that these gender biases endured for several days after the experiment.

A.2.9 Examining the Sources of Online Images

In this supplementary analysis, we examine the online sources from which the images in our sample primarily derive, since the Google Image search engine serves mainly as an intermediary for routing internet users to websites containing particular images. For this purpose, we leveraged data from our main experiment, which required participants to report the website that they downloaded each image from after they initially searched for this image on Google. A trained team of five undergraduates then classified the type of website from which participants downloaded each image; all final classifications used were the result of consensus reached among these annotators. These online sources are classified into nine classes: blogs, entertainment (e.g., IMDb), Governmental, Social Media (e.g. Facebook and Twitter), News, Stock Photos, Business, Educational (e.g. Wikipedia), and unknown.

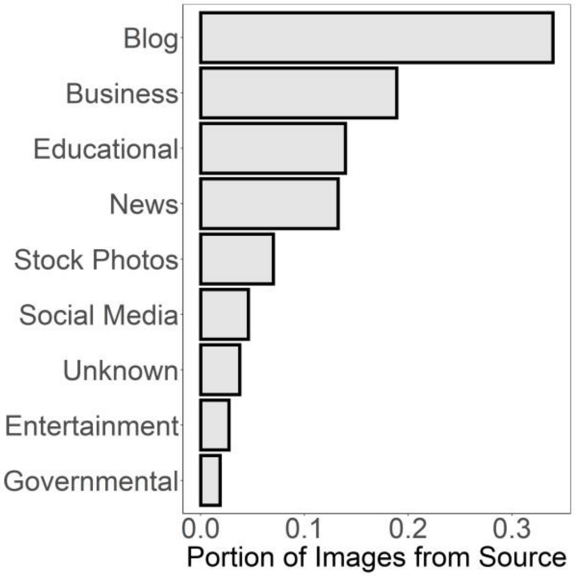

Fig. S24: The distribution of online sources of the images that participants downloaded and uploaded as part of our main experiment.

Fig. S24 presents the distribution of online sources for the images that participants downloaded and uploaded in our main experiment. We find that these images were extracted from a variety of distinct sources; no single type of website supplied the majority of images. The most common source – personal blogs – comprised 34% of images. This suggests that many images in our sample derived from personal websites, where images are typically

selected and uploaded at the discretion of internet users, reflecting a real-world data generation process (i.e., reflecting images that real people elect to use on their personal websites). A related selection process is reflected in the second most popular source of images - business websites - where images are often designed and uploaded as part of marketing materials. News platforms are the fourth most common source (16%), followed by stock photo websites (10%), suggesting that industries characterized by image-editing processes contribute to the data. Based on these results, it is reasonable to suggest that the Google images we examine do, indeed, reflect a variety of real-world data generating processes, which are mediated not only by algorithms, but also by psychological factors motivating the human choice of which content to curate and upload. In other words, despite its limitations, this analysis quite clearly suggests that no single source of images (e.g. "stock photos") appears to be artificially driving our results; that of course would be concerning, since it would suggest that our study is really a study of the market dynamics of the stock photo industry as compared to regular Google text search results. However, we find no evidence to support this concern. Stock photo websites were only identified as the fifth most common source of images. This needs to be taken with a grain of salt, since it is possible for people to purchase and copy stock photo images and reuse them on their personal and professional websites. However, we think this is unlikely to bias our results since less than 5% of images in our experimental data repeated across sources. The same is true in our observational data, where we find that only 11% of images repeat within or across searches, and all of our results are robust to the exclusion of repeat images. Altogether, these findings suggest that the images in our sample derive from a variety of sources that engage a range of distinct, real-world data generating processes, suggesting that the gender bias we observe likely reflects a general bias that seeps into online images through multiple channels and mechanisms.

Supplementary References

[S1] Jeffrey Pennington, Richard Socher, and Christopher D Manning. Glove: Global vectors for word representation. In *Proceedings of the 2014 conference on empirical methods in natural language processing (EMNLP)*, pages 1532–1543, 2014.

[S2] Krishna Srinivasan, Karthik Raman, Jiecao Chen, Michael Bendersky, and Marc Najork. Wit: Wikipedia-based image text dataset for multimodal multilingual machine learning. In *Proceedings of the 44th International ACM SIGIR Conference on Research and Development in Information Retrieval*, pages 2443–2449, 2021.

[S3] Shane Greenstein and Feng Zhu. Is wikipedia biased? *American Economic Review*, 102(3):343–48, 2012.

[S4] Rasmus Rothe, Radu Timofte, and Luc Van Gool. Deep expectation of real and apparent age from a single image without facial landmarks. *International Journal of Computer Vision*, 126(2-4):144–157, 2018.

[S5] Tomas Mikolov, Edouard Grave, Piotr Bojanowski, Christian Puhrsch, and Armand Joulin. Advances in pre-training distributed word representations. *arXiv preprint arXiv:1712.09405*, 2017.

[S6] Yushi Jing and Shumeet Baluja. Pagerank for product image search. In *Proceedings of the 17th international conference on World Wide Web*, pages 307–316, 2008.

[S7] Debbie S Ma, Joshua Correll, and Bernd Wittenbrink. The chicago face database: A free stimulus set of faces and norming data. *Behavior research methods*, 47:1122–1135, 2015.

[S8] Jhan Alarifi, John Fry, Darren Dancey, and Moi Hoon Yap. Understanding face age estimation: humans and machine. In *2019 International Conference on Computer, Information and Telecommunication Systems (CITS)*, pages 1–5. IEEE, 2019.

[S9] Sean A. Munson Matthew Kay, Cynthia Matuszek. Unequal representation and gender stereotypes in image search results for occupations. *CHI '15: Proceedings of the 33rd Annual ACM Conference on Human Factors in Computing Systems*, page 3819–3828, 2015.

[S10] Dana¨e Metaxa, Michelle A Gan, Su Goh, Jeff Hancock, and James A Landay. An image of society: Gender and racial representation and impact in image search results for occupations. *Proceedings of the ACM on Human-Computer Interaction*, 5(CSCW1):1–23, 2021.

[S11] Klaus Krippendorff. *Content analysis: An introduction to its methodology*. Sage publications, 2018.

[S12] Kilem L Gwet. *Handbook of inter-rater reliability: The definitive guide to measuring the extent of agreement among raters*. Advanced Analytics, LLC, 2014.

[S13] Joseph Lilleberg, Yun Zhu, and Yanqing Zhang. Support vector machines and word2vec for text classification with semantic features. In *2015 IEEE 14th International Conference on Cognitive Informatics & Cognitive Computing (ICCI* CC)*, pages 136–140. IEEE, 2015.

[S14] Nikhil Garg, Londa Schiebinger, Dan Jurafsky, and James Zou. Word embeddings quantify 100 years of gender and ethnic stereotypes. *Proceedings of the National Academy of Sciences*, 115(16):E3635–E3644, 2018.

[S15] Tessa ES Charlesworth, Aylin Caliskan, and Mahzarin R Banaji. Historical representations of social groups across 200 years of word embeddings from google books. *Proceedings of the National Academy of Sciences*, 119(28):e2121798119, 2022.

[S16] Robyn Speer, Joshua Chin, and Catherine Havasi. Conceptnet 5.5: An open multilingual graph of general knowledge. In *Proceedings of the AAAI conference on artificial intelligence*, volume 31, 2017.

[S17] Hicham El Boukkouri, Olivier Ferret, Thomas Lavergne, Hiroshi Noji, Pierre Zweigenbaum, and Junichi Tsujii. Characterbert: Reconciling elmo and bert for word-level open-vocabulary representations from characters. *arXiv preprint arXiv:2010.10392*, 2020.

[S18] Tom Brown, Benjamin Mann, Nick Ryder, Melanie Subbiah, Jared D Kaplan, Prafulla Dhariwal, Arvind Neelakantan, Pranav Shyam, Girish Sastry, Amanda Askell, et al. Language models are fewshot learners. *Advances in neural information processing systems*, 33:1877–1901, 2020.

[S19] James Evans Austin Kozlowski, Matt Taddy. The geometry of culture: Analyzing the meanings of class through word embeddings. *American Sociological Review*, 84:905–949, 2019.

[S20] Kawin Ethayarajh. How contextual are contextualized word representations? comparing the geometry of bert, elmo, and gpt-2 embeddings. *arXiv preprint arXiv:1909.00512*, 2019.

[S21] Qianchu Liu, Diana McCarthy, and Anna Korhonen. Towards better context-aware lexical semantics: Adjusting contextualized representations through static anchors. In *Proceedings of the 2020 Conference on Empirical Methods in Natural Language Processing (EMNLP)*, pages 4066–4075, 2020.

[S22] Jun Gao, Di He, Xu Tan, Tao Qin, Liwei Wang, and Tie-Yan Liu. Representation degeneration problem in training natural language generation models. *arXiv preprint arXiv:1907.12009*, 2019.

[S23] Joy Adowaa Buolamwini. *Gender shades: intersectional phenotypic and demographic evaluation of face datasets and gender classifiers*. PhD thesis, Massachusetts Institute of Technology, 2017.

[S24] Shruti Nagpal, Maneet Singh, Richa Singh, and Mayank Vatsa. Deep learning for face recognition: Pride or prejudiced? *arXiv preprint arXiv:1904.01219*, 2019.

[S25] Gary B Huang, Marwan Mattar, Tamara Berg, and Eric Learned-Miller. Labeled faces in the wild: A database for studying face recognition in unconstrained environments. In *Workshop on faces in 'RealLife' Images: detection, alignment, and recognition*, 2008.

[S26] Hu Han, Anil K Jain, et al. Age, gender and race estimation from unconstrained face images. *Dept. Comput. Sci. Eng., Michigan State Univ., East Lansing, MI, USA, MSU Tech. Rep.(MSU-CSE-14-5)*, 87:27, 2014.

[S27] Brendan F Klare, Ben Klein, Emma Taborsky, Austin Blanton, Jordan Cheney, Kristen Allen, Patrick Grother, Alan Mah, and Anil K Jain. Pushing the frontiers of unconstrained face detection and recognition: Iarpa janus benchmark a. In *Proceedings of the IEEE conference on computer vision and pattern recognition*, pages 1931–1939, 2015.

[S28] R. Axelrod R, Iliev. The paradox of abstraction: Precision versus concreteness. *Journal of Psycholinguistic Research*, 46:715–29, 2017.

[S29] Abeba Birhane, Vinay Uday Prabhu, and John Whaley. Auditing saliency cropping algorithms. In *Proceedings of the IEEE/CVF Winter Conference on Applications of Computer Vision*, pages 4051– 4059, 2022.

[S30] Rafael Padilla, CFF Costa Filho, and MGF Costa. Evaluation of haar cascade classifiers designed for face detection. *World Academy of Science, Engineering and Technology*, 64:362–365, 2012.

[S31] M Hassaballah, Kenji Murakami, and Shun Ido. Face detection evaluation: a new approach based on the golden ratio ϕ. *Signal Image Video Process.*, 7(2):307–316, 2013.

[S32] Bin Yang, Junjie Yan, Zhen Lei, and Stan Z Li. Fine-grained evaluation on face detection in the wild. In *2015 11th IEEE International Conference and Workshops on Automatic Face and Gesture Recognition (FG)*, volume 1, pages 1–7. IEEE, 2015.

[S33] William E Hockley. The picture superiority effect in associative recognition. *Memory & cognition*, 36(7):1351–1359, 2008.
