# Supplementary material for: Online images amplify gender bias
Source: Nature. 2024 Feb 14;626(8001):1049–55. doi: 10.1038/s41586-024-07068-x (PMC10901730; doi:10.1038/s41586-024-07068-x)
Supplement: Supplementary file 2 — Reporting Summary [file 41586_2024_7068_MOESM2_ESM.pdf]

nature portfolio

Corresponding author(s): Douglas Guilbeault

Last updated by author(s): Dec 13, 2023

Reporting Summary

Nature Portfolio wishes to improve the reproducibility of the work that we publish. This form provides structure for consistency and transparency in reporting. For further information on Nature Portfolio policies, see our Editorial Policies and the Editorial Policy Checklist.

Statistics

For all statistical analyses, confirm that the following items are present in the figure legend, table legend, main text, or Methods section.

n/a	Confirmed	
☐	☒	The exact sample size (*n*) for each experimental group/condition, given as a discrete number and unit of measurement
☐	☒	A statement on whether measurements were taken from distinct samples or whether the same sample was measured repeatedly
☐	☒	The statistical test(s) used AND whether they are one- or two-sided<br>*Only common tests should be described solely by name; describe more complex techniques in the Methods section.*
☐	☒	A description of all covariates tested
☐	☒	A description of any assumptions or corrections, such as tests of normality and adjustment for multiple comparisons
☐	☒	A full description of the statistical parameters including central tendency (e.g. means) or other basic estimates (e.g. regression coefficient) AND variation (e.g. standard deviation) or associated estimates of uncertainty (e.g. confidence intervals)
☐	☒	For null hypothesis testing, the test statistic (e.g. *F*, *t*, *r*) with confidence intervals, effect sizes, degrees of freedom and *P* value noted<br>*Give P values as exact values whenever suitable.*
☒	☐	For Bayesian analysis, information on the choice of priors and Markov chain Monte Carlo settings
☒	☐	For hierarchical and complex designs, identification of the appropriate level for tests and full reporting of outcomes
☐	☒	Estimates of effect sizes (e.g. Cohen's *d*, Pearson's *r*), indicating how they were calculated

Our web collection on statistics for biologists contains articles on many of the points above.

Software and code

Policy information about availability of computer code

Data collection	All observational data was collected using custom code written in Python by our team (Python version 3.12.0). Experimental data was collected using Qualtrics. The IAT component of the experiment was collected using the iatgen software (version release 1.0), see link here: https://iatgen.wordpress.com/
Data analysis	All data analyses were completed using custom code written in both R and Python by our team. Python version 3.12.0; R version 4.3.2. All data and code relating to data analyses is publicly available at the following github: https://github.com/drguilbe/ImgVSText

For manuscripts utilizing custom algorithms or software that are central to the research but not yet described in published literature, software must be made available to editors and reviewers. We strongly encourage code deposition in a community repository (e.g. GitHub). See the Nature Portfolio guidelines for submitting code & software for further information.

Data

Policy information about availability of data

All manuscripts must include a data availability statement. This statement should provide the following information, where applicable:

- Accession codes, unique identifiers, or web links for publicly available datasets
- A description of any restrictions on data availability
- For clinical datasets or third party data, please ensure that the statement adheres to our policy

All data collected in association with this project is publicly available at the following github: https://github.com/drguilbe/ImgVSText

Field-specific reporting

Please select the one below that is the best fit for your research. If you are not sure, read the appropriate sections before making your selection.

☐ Life sciences ☒ Behavioural & social sciences ☐ Ecological, evolutionary & environmental sciences

For a reference copy of the document with all sections, see nature.com/documents/nr-reporting-summary-flat.pdf

Behavioural & social sciences study design

All studies must disclose on these points even when the disclosure is negative.

Study description	This study has two components. The first component is observational and provides a quantitative algorithmic audit of gender stereotypes in online texts and images (from Google, Wikipedia, and IMDb). The second component is experimental and compares the effects of googling for images or textual descriptions of occupations on participants' explicit and implicit gender bias (specifically on their implicit bias toward associating men with science and women with liberal arts).
Research sample	The research sample for the experimental component of this study consists of a nationally representative sample of the U.S., as curated by the crowdsourcing platform Prolific. The details on Prolific's U.S. nationally representative sample are provided by Prolific at the following link: https://researcher-help.prolific.co/hc/en-gb/articles/360019236753-Representative-samples.<br><br>To create a representative U.S. sample, Prolific takes the intended sample size and stratifies it across three demographics: age, sex and ethnicity. Prolific uses census data from the US Census Bureau to divide the sample into subgroups with the same proportions as the national U.S. population. This means, for example, that a representative sample contains the same proportion of 28-37 year old Asian women as the national population (to the extent possible). Using this representative sample is important for our experiment which does not make any demographic-specific predictions around the effects of internet search modality on gender bias; instead, we aim to identify an effect of internet search modality across demographic groups, and for this reason, using a representative sample enhances our ability to claim that our effect holds in a population whose demographic composition captures the diversity that characterizes the entire U.S. population, and is not, therefore, an artifact of an idiosyncratic demographic distribution in our sample.
Sampling strategy	For the experimental component of our study, our sampling strategy was a random sample from Prolific's nationally representative panel (N=600, see "Research sample"). No statistical methods were used to determine sample size prior to data collection. We could not find any prior work that could provide a reasonable estimate of the expected effect size needed to develop robust power calculations. Given that the participants in our sample were statistically independent and our between-condition comparisons used basic WIlcoxon/Student T tests, 150 subjects per condition was deemed to be more than sufficient, since these tests can readily return robust, statistically significant differences between conditions for sample sizes of less than 30 per condition. Moreover, given that participants in our experiment rated 22 occupations, our experiment provided 13,200 raw estimates, such that we expected regression analyses using the raw data -- as well as within-subject analyses -- would be more than sufficiently powered. The experiment presented in this published paper is a replication of a prior experiment which used the same sample size and provided clear results (pre-registration here: https://osf.io/26kbr), so we maintained this sample size in our successful replication (which differed only by the inclusion of a control condition).<br><br>For the observational component of this study, our sample of Google Images was collected through the following standardized procedure. We started by using each of the social categories in Wordnet to automatically search and retrieve the top 100 images in Google corresponding to each social category in Google Images (Google provides roughly 100 images by default for its initial results on a given search query). Each search was implemented from a fresh Google account with no prior history to avoid the uncontrolled effects of Google's recommendation algorithm, which customizes search results based on browsing history. Searches were run by 10 distinct data servers in New York City. All image data from Google was collected in August 2020. For the sample of Google News articles, we used the pre-trained word2vec embedding models of over 100 billion word tokens from Google News as provided by the gensim Python package: https://code.google.com/archive/p/word2vec/. In total, we compare our image data (including data from Wikipedia) against seven popular word embedding models (see Table S1 in our appendix). For each category, we extracted the publicly available images that Wikipedia provides on the Wikipedia article corresponding to this category. All images from Wikipedia were extracted using WIT ("Wikipedia Image Text Dataset"), the largest multimodal dataset on record (to date). WIT was released in 2021 and can be accessed here: https://github.com/google-research-datasets/wit
Data collection	The online textual data is derived from publicly available repositories of online articles from Google News and Wikipedia. The online image data is similarly derived from publicly available image repositories stored via Google Image Search and Wikipedia (see "Sampling strategy"). The experimental data collection was implemented using a survey instrument designed in Qualtrics; for more information.
Timing	All of our main image data from Google was collected in August 2020 (see "Sampling strategy"). All textual data from Google news is based on publicly available embedding models via the gensim Python package, which were first released in 2013 and were updated in 2021. All images from Wikipedia were extracted using WIT ("Wikipedia Image Text Dataset"), which was released in 2021.
Data exclusions	No data were excluded from observational analyses. For our experimental design, we only examined data associated with recruited participants who successfully joined and completed the task. 575 of the 600 participants recruited to our study completed the task, exhibiting an attrition rate of 4.2%.
Non-participation	No participants dropped out of the experiment.

Randomization	In the experimental component of our study, participants were randomized to one of four conditions: (1) the Image condition, (2) the Google News Text condition, (3) the Generic Google Search Bar Text condition, and (4) the Control condition.

Reporting for specific materials, systems and methods

We require information from authors about some types of materials, experimental systems and methods used in many studies. Here, indicate whether each material, system or method listed is relevant to your study. If you are not sure if a list item applies to your research, read the appropriate section before selecting a response.

Materials & experimental systems

n/a	Involved in the study	
☒	☐	Antibodies
☒	☐	Eukaryotic cell lines
☒	☐	Palaeontology and archaeology
☒	☐	Animals and other organisms
☐	☒	Human research participants
☒	☐	Clinical data
☒	☐	Dual use research of concern

Methods

n/a	Involved in the study	
☒	☐	ChIP-seq
☒	☐	Flow cytometry
☒	☐	MRI-based neuroimaging

Human research participants

Policy information about studies involving human research participants

Population characteristics	The research sample for the experimental component of this study consists of a nationally representative sample of the U.S., as curated by the crowdsourcing platform Prolific. The details on Prolific's U.S. nationally representative sample are provided by Prolific at the following link: https://researcher-help.prolific.co/hc/en-gb/articles/360019236753-Representative-samples. The sample population is representative of the national US population along the following demographic variables: age, sex and ethnicity.
Recruitment	For the experimental component, our sampling strategy was a random sample from Prolific's nationally representative panel (N=600, see "Research sample").
Ethics oversight	This research was approved by the Institutional Review Board at the University of California, Berkeley, where the study was run.

Note that full information on the approval of the study protocol must also be provided in the manuscript.
